# Convergent evolution in *Arabidopsis halleri* and *Arabidopsis arenosa* on calamine metalliferous soils

**DOI:** 10.1098/rstb.2018.0243

**Published:** 2019-06-03

**Authors:** Veronica Preite, Christian Sailer, Lara Syllwasschy, Sian Bray, Hassan Ahmadi, Ute Krämer, Levi Yant

**Affiliations:** 1 Molecular Genetics and Physiology of Plants, Faculty of Biology and Biotechnology, Ruhr University Bochum, 44801 Bochum, Germany; 2 Cell and Developmental Biology, John Innes Centre, Norwich NR4 7UH, UK; 3 School of Life Sciences, University of Nottingham, Nottingham NG7 2RD, UK

**Keywords:** convergence, adaptation, evolution, selective sweep, selection

## Abstract

It is a plausible hypothesis that parallel adaptation events to the same environmental challenge should result in genetic changes of similar or identical effects, depending on the underlying fitness landscapes. However, systematic testing of this is scarce. Here we examine this hypothesis in two closely related plant species, *Arabidopsis halleri* and *Arabidopsis arenosa*, which co-occur at two calamine metalliferous (M) sites harbouring toxic levels of the heavy metals zinc and cadmium. We conduct individual genome resequencing alongside soil elemental analysis for 64 plants from eight populations on M and non-metalliferous (NM) soils, and identify genomic footprints of selection and local adaptation. Selective sweep and environmental association analyses indicate a modest degree of gene as well as functional network convergence, whereby the proximal molecular factors mediating this convergence mostly differ between site pairs and species. Notably, we observe repeated selection on identical single nucleotide polymorphisms in several *A. halleri* genes at two independently colonized M sites. Our data suggest that species-specific metal handling and other biological features could explain a low degree of convergence between species. The parallel establishment of plant populations on calamine M soils involves convergent evolution, which will probably be more pervasive across sites purposely chosen for maximal similarity in soil composition.

This article is part of the theme issue ‘Convergent evolution in the genomics era: new insights and directions’.

## 1. Introduction

Most plants cannot rapidly escape hostile environments. Thus, they present powerful models for the study of adaptation. Remarkably, some plant species contain multiple populations that have evolved the ability to thrive in the harshest environments, for example extreme drought, solar radiation, heat, salinity, low nutrient availability and toxic concentrations of heavy metal ions in soil. Metalliferous (M) soils are defined as rich in at least one class B and borderline trace metal element [1], are usually nutritionally imbalanced [2,3], and arise either through geological (e.g. ancient outcrop) or human (e.g. mining, metal smelter) activity. Such soils are generally toxic to plants and host a sparse, species-poor characteristic vegetation of adapted, often endemic extremophiles, so-called metallophytes [4].

Several members of the *Arabidopsis* genus have been described as pseudo-metallophytes, i.e. harbouring populations on both M and nonmetalliferous (NM) soils, namely *Arabidopsis halleri* [5,6], *Arabidopsis arenosa* [7–9] and *Arabidopsis lyrata* [10]. Among these species, only *A. halleri* is widespread on calamine-type M soils of Central and Eastern Europe as well as in East Asia and has thus become a model organism for the study of evolutionary adaptation to challenging soils. Calamine soils are defined as containing high levels of zinc (Zn), which are geologically accompanied by the metals cadmium (Cd), lead (Pb) and occasionally copper (Cu). *Arabidopsis halleri* is a diploid (2*n* = 16), stoloniferous perennial and obligate outcrosser with a haploid genome size of approximately 260 Mbp [11]. On both M and NM soils, *A. halleri* exhibits Zn, and regionally also Cd, hyperaccumulation, defined as the ability to accumulate greater than 3000 µg Zn g^−1^ dry leaf biomass or greater than 100 µg Cd g^−1^ dry leaf biomass in its natural habitat [5,6]. Experimental studies in synthetic hydroponic media have demonstrated species-wide hypertolerance to both metals in comparison to the closely related species *A. lyrata* and *Arabidopsis thaliana* [12,13]. These same studies also established that *A. halleri* accessions originating from calamine M soils exhibit enhanced Zn and/or Cd hypertolerance, which is probably the result of local adaptation. Importantly, the basal metal tolerance present in all plants, which enables them to acclimate to local fluctuations in soil composition, does not allow survival on calamine M soils [14].

*Arabidopsis arenosa* occurs as diploid (2*n* = 16) and autotetraploid (2*n* = 32) cytotypes and is a perennial obligate outcrosser with ample seed set in the field, compared with *A. halleri*. Generally, *A. arenosa* is absent from most calamine M soils and is known as a so-called metal excluder, i.e. a plant that maintains normal Zn and low Cd concentrations in its above-ground biomass in natural populations [15,16]. While *A. halleri* and *A. arenosa* generally occupy differing edaphic niches, both species are rarely found together at few calamine M sites [17] in Eastern Europe and at some NM sites, suggesting that their populations can occasionally undergo convergent niche shifts. Our information on tolerance to and accumulation of calamine-type metals in *A. arenosa* and its intra-species variation is still rudimentary [7,8,18,19].

Work over the past two decades has established a first understanding of the genetic basis of species-wide metal hypertolerance and hyperaccumulation in *A. halleri* in comparison to closely related species [11,20,21]. However, no causal genetic locus governing within-species variation in tolerance to calamine-type metals has been identified to date. With this study, we aimed to detect convergent genomic footprints of selection at two calamine M sites, each by comparison to a NM site in their vicinity, in both *A. halleri* and *A. arenosa*. We thus took advantage of a few exceptional cases where both species have adapted to the same sites and thus similarly composed soils. Individual genome resequencing of 64 individual plants from eight populations, followed by high-density genome scans for selective sweeps, identified a handful of compelling candidate genes under selection at both of the two site pairs or in both of the two species. Notable among these, we identify the *A. halleri Cysteine Protease-Like 1 (CPL1)* locus as a candidate for convergent selection in both population pairs. We show that this gene exhibits a series of convergent derived sequence variants in individuals originating from M sites, and appears to have undergone a loss of function in populations at NM sites.

## 2. Methods

### (a) Field sampling, and plant and soil materials

Field sampling was conducted for multi-element analysis from *A. halleri* (L.) O'Kane and Al-Shehbaz ssp. *halleri* and *A. arenosa* ssp*. arenosa* (L.) Hayek at four field sites in September 2015 and May 2016 (seven to 10 individuals per species and site; see the electronic supplementary material, table S1). From each plant individual, we collected both a sample of root-proximal soil for multi-element analysis and a leaf sample for DNA extraction (see [6] for a description of sites and methods for soil sample collection and processing). Five to 10 leaves per individual were placed in a 2 ml polypropylene tube for later DNA isolation, immediately frozen in liquid nitrogen (MVE vapor shipper, Chart, Minnesota, USA) and stored in liquid nitrogen. For experiments under controlled growth chamber conditions, we collected about 40 l of soil (≤0.3 m depth) at the M site Miasteczko Śląskie (Mias; see the electronic supplementary material, table S1) in May 2016 (see below). All-purpose greenhouse soil (Minitray, Einheitserde, Sinntal-Altengronau, Germany) was used as NM control soil. Plants were grown from cuttings of *A. halleri* individuals collected at Mias and Zakopane [6] and maintained in the greenhouse (Ruhr-Universität Bochum, Germany, [6]) and from *A. arenosa* seeds collected at the two field sites Mias and Zakopane (Zapa).

### (b) Plant cultivation under growth chamber conditions

Seedlings of *A. arenosa* and vegetative clones of *A. halleri* (see the electronic supplementary material, Supplementary methods, a) were pre-cultivated for 17 days in 1 : 1 (v/v) peat : sand (round pots, 5 cm Ø, 3.5 cm depth, 50 ml volume) in a climate-controlled growth chamber (20°C/17°C, 10 h light at 100 µmol m^−2^ s^−1^; GroBanks, Arabidopsis BB-XXL.3, CLF Plant Climatics, Wertingen, Germany). Subsequently, plants were transferred into experimental treatment soils (three volume parts of field-collected M Mias or NM greenhouse soil, each mixed with one volume part of sand; square pots 7 × 7 cm width, 8 cm depth, 300 ml volume) and cultivated in the growth chamber for another six weeks. Pots were arranged in trays (separated by species and treatment soil; 16–23 pots per tray), and plants were watered with tap water (poured from above) when needed (two to three times per week, preventing waterlogging). Positions and orientation of trays were re-arranged randomly once per week. Photographs were taken at the start, after three weeks and at the end of cultivation. At harvest, plant survival was scored and fresh above-ground biomass was determined for each plant. The biomass between experimental groups was assessed using a generalized linear mixed effect model with fresh biomass as dependent variable, genotype (site of origin: Mias M site versus Zapa NM site) and treatment (experimental soil type of exposure: control-soil versus M soil) as fixed predictors. Individual plants and genotype nested in treatment were set as random factors. The significance of each variable as well as the interaction between genotype and treatment was tested with type II *χ*^2^ based likelihood-ratio tests (based on inverse Gaussian distribution with the link function 1/mu^2^; glmer and ANOVA functions in R-packages lme4 [22] and car [23], respectively).

### (c) Analysis of soil samples and DNA extraction

Soil pH, as well as extractable and exchangeable concentrations of Al, B, Ca, Cd, Cr, Cu, Fe, K, Mg, Mn, Ni, P, Pb, S and Zn in soil samples were determined as described [6]. Frozen leaf tissues were lyophilized overnight (Alpha 1-4 LSC plus, Martin Christ LCG, Osterode am Harz, Germany) and subsequently homogenized with a single ceramic bead (3 mm Ø; Precellys Beads, Peqlab, Erlangen, Germany) in a Retsch mixer mill (Type MM300, Retsch, Haan, Germany) for 2 × 1.5 min at 30 Hz. For each individual sample 9–15 mg of dry leaf powder was weighed into a 2 ml polypropylene tube and mixed thoroughly with 0.9 ml cetyl trimethylammonium bromide buffer, followed by DNA extraction according to [24] with small modifications (see the electronic supplementary material, Supplementary methods). DNA quality was verified by spectrophotometry and agarose gel electrophoresis, and DNA was quantified using the dsDNA HS assay (Q32854) following the manufacturer's instructions with an incubation time of 20 min (Qubit 3.0, ThermoFisher Scientific, Life Technologies Ltd., Paisley, UK).

### (d) Library preparation, sequencing, processing of next generation sequencing data, and variant calling

We prepared Illumina TruSeq polymerase chain reaction (PCR) free (FC-121-3003; Illumina United, Fulbourn, UK) sequencing libraries with 350 bp insert lengths according to manufacturer's instructions with slight modifications. We processed the sequencing data files using custom Python3 or Bash scripts that allowed batch processing on high performance cluster computers. Workflows were based on GATK Best Practices, GATK version 3.6 or higher [25]. The next generation sequencing (NGS) data processing pipeline involved initial processing of raw sequence data, mapping, re-aligning of sequence data around indels, and variant discovery (electronic supplementary material, Supplementary Methods, https://github.com/syllwlwz/Divergence-Scans/tree/master/SNP_calling_arenosa). For each species, we mapped sequence reads to the high quality chromosome-build *A. lyrata* reference genome (JGI Phytozome, https://phytozome.jgi.doe.gov/pz/portal.html). Mapping both species to the same reference allows a clean comparison of the same gene space in both species. However, given this design our study cannot reliably assess regions of the genomes of *A. halleri* or *A. arenosa* that are highly divergent from the genome of *A. lyrata*. The genome assemblies presently available for *A. arenosa* and *A. halleri* are of insufficient quality for conducting genome scans, thus outweighing any disadvantage arising from mapping reads to the heterologous *A. lyrata* genome. Neutral population structure was assessed based on initial NGS data processing as conducted for environmental association analysis (EAA), employing the putatively neutral fourfold degenerate sites from the filtered variant call files. We extracted the allele frequency per individual and used this data for a principal component analysis (PCA) using the R-package FactoMineR [26].

### (e) Genome scans, large-effect variant identification, candidate gene lists and test for convergent evolution

For each population pair, the genome was partitioned into windows of 25 consecutive single nucleotide polymorphisms (SNPs), for which we calculated the per-window mean of each pairwise metric diversity-divergence residuals (DD) [9,27], Wright's fixation index (*F*_ST_) [9,28,29], a two-dimensional site frequency spectrum composite likelihood ratio test (Nielsen 2dSFS) [9,27,30], absolute net divergence (*d_XY_*) [9,31], the Lewontin–Krakauer LK test (Flk) [32], VarLD [33], absolute allele frequency difference (AFDabs), the single population metrics Tajima's *D* [34] and Fay and Wu's *H* [35], as well as SweeD [30,36]. We proceeded in this manner in order to reduce sampling noise of single SNPs and to avoid the known caveats of windows of set length in base pairs [37]. We used different metrics to address different ages of selection events and corresponding divergence times [38]. Candidate windows for selection were identified as ≥99.9%iles (≤0.1%ile for DD) of all windows for either one of the pairwise metrics for each population contrast. Orthologous *A. thaliana* gene identifiers were retrieved for *A. lyrata* genes and genes not assigned to an OrthoGroup were submitted for a local blastx on the nr database. All candidate genes underwent a custom annotation process for filtering. All variants (SNPs and indels) were annotated and their effects predicted by SnpEff [39] based on the *A. lyrata* annotation version 2 [40]. SnpEff uses the reference annotation to predict the effects of variants on the encoded proteins.

In addition to the genome scans described above, we used the genome-wide output of SnpEff to identify large-effect variants at divergent frequencies between populations of a pair. This identified putative candidate genes of high allele frequency difference between M and NM populations, which may have escaped detection in genome scans. Coverage was calculated per gene or per exonic gene content and the number of paralogous genes within the same OrthoGroup was extracted. To identify candidate genes shared between site pairs or between species, Venn diagrams of candidate genes were generated with Venny 2.1.0 [41], and hypergeometric tests were performed to compare observed and expected overlaps. A gene function enrichment test was performed for each population pair using the ClueGO app version 2.5.2 [42] in Cytoscape version 3.6.1 [43] using the *A. thaliana* gene identifiers and the gene ontology (GO) ‘BiologicalProcess’.

### (f) Identifying divergence signatures, environmental association analysis, and compilation of candidate gene lists

To identify divergence signatures, we calculated the per-window mean values for allele frequency difference (AFD), *d_XY_* [9,31], *F*_ST_ [9,28,29], DD [9,27], and Tajima's *D* [34] in SNP based windows. We defined a divergence signature as ≥99.5%ile windows in the empirical distributions for each metric (https://github.com/SailerChristian/Divergence_Scan). If at least one of the metrics *d_XY_*, *F*_ST_, or DD showed a divergence signature overlapping a gene, we considered this gene as a DivergenceScan candidate.

Signatures of positive selection are expected to harbour a divergence signature and an association with a particular selection pressure, in this case we focus on soil trace metal element (TME) concentrations. To identify which of the DivergenceScan candidate genes fulfil the second condition of association with soil TME, we used the environmental association analysis tool Bayenv2 [44], which allows for testing in a two-step process. We therefore took all SNPs overlapping candidate genes identified using DivergenceScan and subsequently used EAA to analyse these for association with extractable Cd and extractable Zn concentrations in soil across all four populations. In order to infer effects on protein structure, we selected environmentally very strongly associated SNPs (environmental associated (EA) SNPs, Bayes Factor (BF) ≥ 100 [45]) that cause a non-synonymous change according to the annotation using SnpEff [39]. To draw conclusions about convergence, we created a union list of extractable Cd and extractable Zn EA SNPs per contrast and species and identified the intersect between the two contrasts within each species.

### (g) Homology modelling and re-assessment of putative intron–exon boundaries

Homology models of *Ah*CPL1 were generated with Modeller 9.20 [46] using the structures of *Actinidia chinensis* (PDB: 2ACT), *Tabernaemontana divaricata* (PDB: 1IWD), *Zingiber officinale* (PDB: 1CQD) and *Homo sapiens* (PDB: 1BY8, identified using PSIPRED [47] and incorporated to model the pro-peptide). The final model was determined based on DOPE score. Multiple sequence alignments were generated using Clustal Omega (https://www.ebi.ac.uk/Tools/msa/clustalo/) and Modeller 9.20 [46], with colour scheme showing percentage identity through Jalview [48].

Intron–exon boundaries were initially determined by alignment to the annotated *A. lyrata* reference genome. Boundaries were re-defined using the *A. halleri* reference genome [49]. *Arabidopsis halleri* introns from gene Araha.2668s0004 were aligned to the genomic consensus sequences of *Ah*CPL1. In this way, intron 2 was expanded by six nucleotides at the 3′ end (2 amino acids) and intron 4 was expanded at the 5′ end by 117 nucleotides for the M site allele and 185 nucleotides for the NM site allele.

## 3. Results

### (a) Choice of site pairs and edaphic characterization of sites and microhabitats

**Figure 1.**
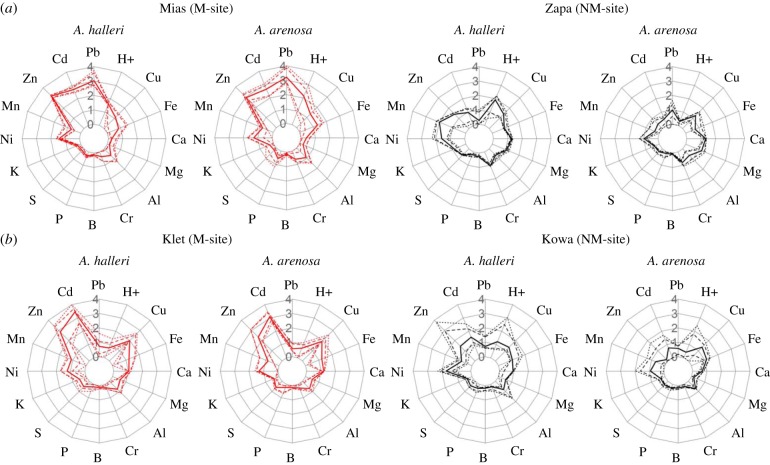
Mineral composition of exchangeable fraction of soils, and soil pH. (*a*) Site pair Miasteczko Śląskie (Mias) and Zakopane (Zapa), (*b*) Site pair Kletno (Klet) and Kowary (Kowa). Concentrations of elements were determined in 0.01 M BaCl_2_ extracts of soils collected in the field directly adjacent to roots of the plant individuals that we resequenced (see Methods). Concentrations (mg element kg^−1^ dry soil mass) were normalized to the global minimum per site pair across both species, and subsequently log_10_-transformed. Shown are the median (solid line), 10 and 90%iles (dashed lines), minimum and maximum (dotted lines) for each site per species (*n* = 5 to 9 plant individuals) for metalliferous (M, red) and non-metalliferous (NM, black) soils.

While sampling *A. halleri* at 165 European sites [6], we noticed the additional presence of *A. arenosa* plants at a small subset of M and NM *A. halleri* sites. According to soil multi-element analysis, *A. halleri* grows in highly metal-contaminated soil patches at M sites whereas *A. arenosa* typically occupies low-metal soil microhabitats (data not shown). However, at Miasteczko Śląskie/PL (Mias) and Kletno/PL (Klet), individuals of both species grew in M soil microhabitats of highly similar composition (figure 1; electronic supplementary material, tables S1 and S2 and dataset S1). Between the two species, the only significant differences were higher extractable Al and extractable Cu concentrations in *A. halleri*-adjacent soil at Klet (*A. halleri* 513 ± 99 mg Al kg^−1^ soil, *A. arenosa* 384 ± 131 mg Al kg^−1^ soil, *F*_1,16_ = 4.930, *p* < 0.05; *A. halleri* 53.25 ± 30.84 mg Cu kg^−1^ soil, *A. arenosa* 27.72 ± 14.53 mg Cu kg^−1^ soil, *F*_1,16_ = 5.514, *p* < 0.04; electronic supplementary material, table S2).

Based on these two focal M sites that were very high in soil exchangeable Zn and Cd, we chose geographically proximal NM sites that also hosted both species, namely Zakopane/PL (Zapa) and Kowary/PL (Kowa), respectively. Plant-proximal soils at these NM sites contained tenfold less or lower average exchangeable soil Zn and Cd concentrations (figure 1). Indeed, exchangeable soil cadmium and zinc concentrations differentiated M from NM sites for both species, based on one-way ANOVA (linear model; Cd: *F*_3,26_ = 149.7, *p* = 2.2 × 10^−16^; Zn: *F*_3,26_ = 68.63, *p* = 1.8 × 10^−12^; electronic supplementary material, table S3). These major global contrasts between M and NM soils were also evident in the extractable fraction of soils (electronic supplementary material, figure S1). Thus, we were able to address the parallel evolution of edaphic adaptation to calamine M soil through the comparison between the site pairs Mias-Zapa and Klet-Kowa in both species, as well as through the comparison between species for either of the two site pairs. NM sites were generally lower in soil pH and higher in soil Zn, Cd and Ni at *A. halleri* microhabitats compared to those of *A. arenosa*.

### (b) Experimental test for adaptation to metalliferous soil

**Figure 2.**
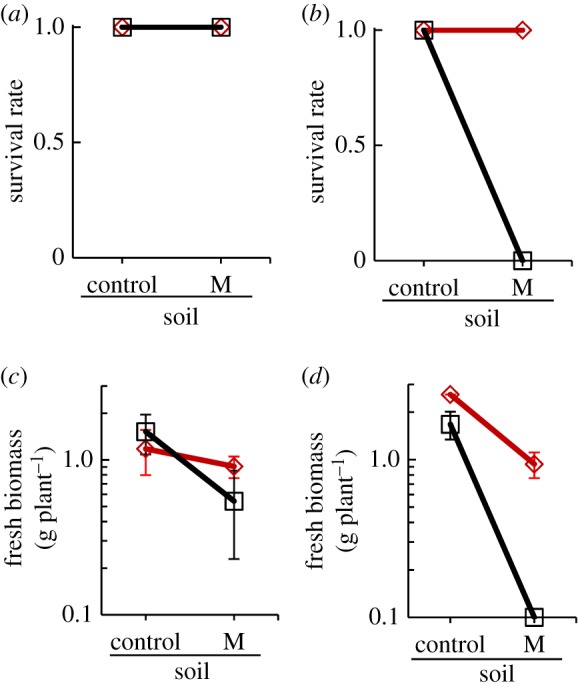
Experimental test for adaptation to metalliferous soil. (*a*,*b*) Survival of *A. halleri* (*a*) and *A. arenosa* (*b*) plants originating from Mias (M site, red) and Zapa (NM site, black) transferred into metalliferous (Mias) or non-metalliferous (control) soil. (c,*d*) Fresh biomass of *A. halleri* (*c*) and *A. arenosa* (*d*) plants originating from Mias (M site, red) and Zapa (NM site, black) transferred into metalliferous (Mias) or non-metalliferous (control) soil. Shown are means and standard deviations of survival rate (*a*,*b*) and fresh above-ground biomass (*c*,*d*) after six weeks of cultivation on experimental soils (see the electronic supplementary material, table S4 for details).

Under climate-controlled growth chamber conditions survival of *A. halleri* was 100% irrespective of plant origin and experimental soil treatment (figure 2*a*; electronic supplementary material, figure S2 and table S4). Similarly, there was 100% survival of *A. arenosa* plants on control soil irrespective of plant origin. However, on M Mias soil, only *A. arenosa* of Mias origin were able to survive (100% survival rate), whereas all plants originating from the NM Zapa site died (survival rate 0%) (figure 2*b*). Biomass production of *A. halleri* exhibited crossing reaction norms, which indicates local adaptation of Mias plants to M Mias soil (figure 2*c*, electronic supplementary material, figure S2, significant interaction between plant genotype and treatment soil at *p* = 0.013, electronic supplementary material, table S4). Similarly, *A. arenosa* exhibited a signature of local adaptation (figure 2*d*, *p* < 0.001). The observed trends were reproduced in an independent experiment (electronic supplementary material, figure S3 and table S4).

### (c) All population pairs are genetically distinct

To assess population structure, we conducted a PCA using putatively neutral (fourfold degenerate) sites. We found that the *A. halleri* individuals from Mias (M1) and Zapa (NM1) were genetically more closely related to one another than those at Klet (M2) and Kowa (NM2). By contrast, in *A. arenosa* we observed greater genetic similarity between the Klet and Kowa populations than between the Mias and Zapa populations (electronic supplementary material, figure S4*a*,*b*). Furthermore, for *A. halleri* the first principal component separated individuals from Klet from those at the other three populations (electronic supplementary material, figure S4*a*). This is most likely caused by a relative excess of low frequency variants in Klet, as illustrated by folded site frequency spectra (electronic supplementary material, figure S4*e*), and consistent with a population bottleneck at Klet. Importantly, the neutral population structure was clearly distinct from the dominant contrast between M and NM soil types in both species.

### (d) Population pairwise identification of candidate genes for selection at metalliferous sites

**Figure 3.**
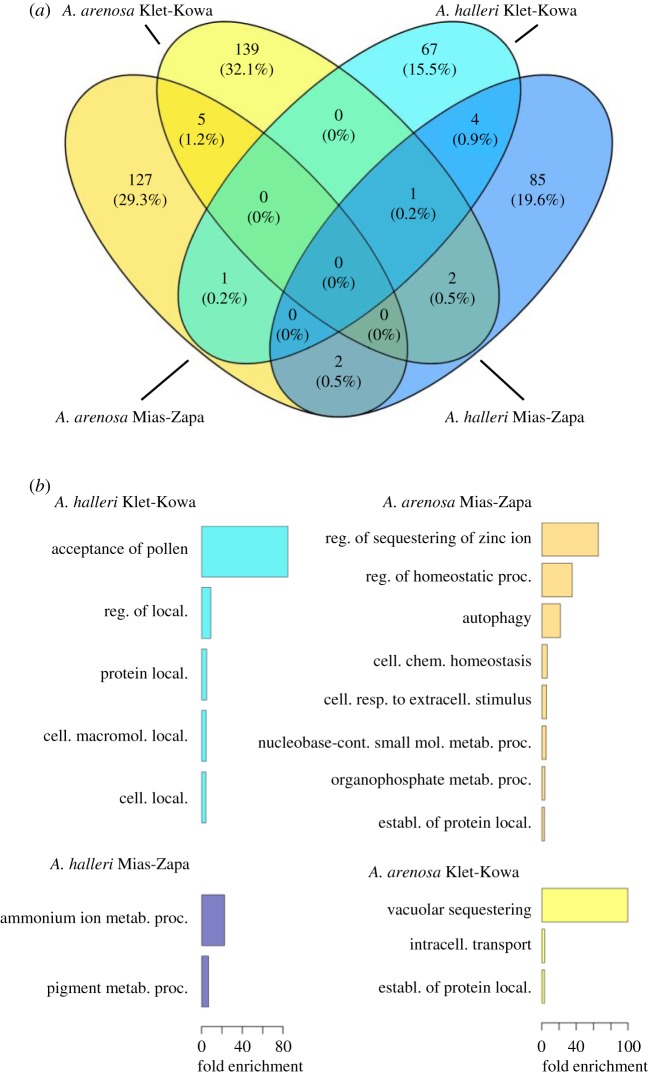
Candidate genes exhibiting signatures of selective sweeps, and functional enrichment analysis. (*a*) Venn diagram shows the number of genes for each population pair in *A. halleri* and in *A. arenosa*, and their intersecting sets. Numbers in parentheses represent the proportion in per cent of all candidate genes shown in the diagram. Candidate genes were among the ≥99.9%iles for any one pairwise genome scan metric and subsequently filtered manually as described in Methods. (*b*) Gene ontology (GO) biological process annotations enriched candidate genes for each population pair. Shown are GO biological processes (levels 1–3) with at least threefold over-representation (*p* < 0.05) among the same candidates as in (*a*) in comparison to the genome-wide average based on *A. thaliana* orthologues for Mias-Zapa in *A. halleri* (dark blue), in *A. arenosa* (dark yellow), Klet-Kowa in *A. halleri* (light blue) and in *A. arenosa* (light yellow).

To obtain candidate loci underlying repeated adaptation to M soils, we next scanned genomes sampled from these populations for selective sweep signatures. We determined gene content of candidate 25 SNP windows based on unique *A. lyrata* gene identifiers, and filtered candidate gene-coding loci manually (see the electronic supplementary material, Supplementary Methods, f). In *A. halleri*, in the single population pair of Mias (M1) and Zapa (NM1) alone, this identified 94 candidate loci based on any one metric (0.1% upper or lower outliers of DD, *F*_ST_, 2dSFS, *d_XY_*, AFDabs or Flk; see Methods) in Mias relative to Zapa (figure 3*a*, dark blue oval; electronic supplementary material, dataset S2). Independently, we identified an additional 81 genes exhibiting predicted large-effect SNPs and 74 genes exhibiting predicted large-effect indels (see Methods; not shown; electronic supplementary material, dataset S2). In the single population pair Klet (M2) by comparison to Kowa (NM2), we identified 73 candidates in genome scans (figure 3*a*, light blue oval), as well as 488 genes containing large-effect SNPs and 379 genes containing large-effect indels (not shown; electronic supplementary material, dataset S3).

In *A. arenosa* for the single population pair Mias-Zapa, we identified 135 candidate genes in divergence window-based scans (figure 3*a*, dark yellow oval), and 15 and 33 genes containing predicted high-effect SNPs and indels, respectively (not shown; electronic supplementary material, dataset S4). Finally, in *A. arenosa* at Klet compared to Kowa, we identified 147 candidate genes (figure 3*a*, light yellow window), as well as five and 16 genes containing high-effect SNPs and indels, respectively (not shown; electronic supplementary material, dataset S5). We conducted an enrichment analysis on GO ‘biological pathways’ with Cytoscape for the candidate genes identified (see Methods; electronic supplementary material, datasets S2–S5). In *A. halleri*, this identified an over-representation among candidates in the functions ‘ammonium ion metabolic process’ for Mias (versus Zapa) and ‘acceptance of pollen’ for Klet (versus Kowa), among others (figure 3*b*). In *A. arenosa*, ‘regulation of sequestering of zinc ion’ were most over-represented among candidate genes at Mias (versus Zapa), and ‘vacuolar sequestering’ for Klet (versus Kowa) (figure 3*b*).

### (e) Degree of convergent evolution

Based on the candidate genes identified in genome scans (§3d above), we identified intersecting sets of genes which represent candidate genes undergoing convergent selection. We identified five candidate genes exhibiting selective sweep signatures that were convergent between both population pairs Mias (versus Zapa) and Klet (versus Kowa) in *A. halleri* (figure 3*a*, intersection of dark blue and light blue oval), and another five convergent candidate genes in *A. arenosa* (figure 3*a*, intersection of dark yellow and light yellow ovals; both *p* < 0.001, hypergeometric test; table 1). There was no convergent candidate gene across both site pairs common to both species (see the electronic supplementary material, dataset S6 for a less conservative list that additionally includes those candidate genes identified through the presence of large-effect SNPs or indels at high allele frequencies). One of the convergent candidate genes across both site pairs in *A. halleri* (AL8G20240, annotated as tRNA dihydrouridine synthase, see table 1 and electronic supplementary material, figure S5*c*) was also a candidate for selection in Klet (versus Kowa) in *A. arenosa*. Two candidate genes were in common between the two species at Mias (versus Zapa), and one gene at Klet (versus Kowa) (n.s., hypergeometric test). Three genes were candidates at Mias in *A. halleri* and at Klet in *A. arenosa* (*p* < 0.01), and one gene was a candidate at Mias in *A. arenosa* and at Klet in *A. halleri* (n.s.). Additionally, there was some convergence among gene functional categories related to cellular protein localization (figure 3*b*). These were significantly over-represented at Klet (versus Kowa) in *A. halleri* and at both site pairs in *A. arenosa* (protein localization, establishment of protein localization, regulation of protein localization, cellular macromolecule localization, cellular localization, intracellular transport; figure 3*b*).

**Table 1.** Convergent candidate genes for selection as identified in this study.

*A. lyrata* genome identifier	*A. thaliana* genome identifier	short gene name	gene annotation^b^
*Arabidopsis halleri* candidate genes convergent between site pairs			
AL2G22120	AT1G65430	ARI8	Ariadne 8; ubiquitin protein ligase^s^
AL2G31400	AT1G71820	SEC6	exocyst complex gene family member; vesicle secretion
AL5G40920	AT3G59040		tetratricopeptide repeat (TPR)-like superfamily protein
AL5G40930	AT2G43020	PAO2	polyamine oxidase 2^s^
AL8G20240	AT5G47970		tRNA dihydrouridine synthase; Aldolase-type TIM barrel^a^
*Arabidopsis arenosa* candidate genes convergent between site pairs			
AL2G12590	AT1G63010	PHT5;1	major facilitator superfamily, SPX domain; vacuolar Pi sequestration^s^
AL2G30820	AT1G71210		pentatricopeptide repeat (PPR) superfamily protein^g,r^
AL7G17030	AT4G34260	AXY8	altered xyloglucan 8; 1,2-α-L-fucosidase; mutant is Al-tolerant^g,r^
AL8G14060	AT5G45140	NRPC2	nuclear RNA polymerase C2^g,f^
AL7G35050	AT4G19050		NB-ARC protein; GWAS association with H_2_O_2_ tolerance
candidate genes at Mias (versus Zapa) convergent between species			
AL5G26930	AT3G47640	PYE	Popeye; bHLH TF acting in iron homeostasis^a^^,g^
AL5G26940	AT3G47650	BDS2	bundle sheath defective 2; DnaJ/Hsp40 cys-rich domain^a^^,l^
candidate genes at Klet (versus Kowa) convergent between species			
AL8G20240	AT5G47970		tRNA dihydrouridine; Aldolase-type TIM barrel family^a^
candidate genes convergent between *A. halleri* at Mias (versus Zapa) and *A. arenosa* at Klet (versus Kowa)			
AL1G26370	AT1G14470		pentatricopeptide repeat (PPR) superfamily protein^f,g^
AL8G20240	AT5G47970		tRNA dihydrouridine; Aldolase-type TIM barrel family^a^
AL6G35310	AT5G23980	FRO4	Fe reduction oxidase 4; root surface Cu(II) chelate reductase^r,l^
candidate genes convergent between *A. halleri* at Klet (versus Kowa) and *A. arenosa* at Mias (versus Zapa)			
AL1G53790	AT1G47560	SEC3B	exocyst complex gene family member; vesicle secretion
*A. halleri* candidate genes convergent between site pairs identified by EAA			
AL1G34900	AT1G21722		unknown transmembrane protein^f^
AL3G38930	AT3G24250		glycine-rich protein^s^
AL3G53910	AT2G20800	NDB4	NAD(P)H dehydrogenase B4^p^
AL5G24630	none		Blast: Alpha-D-xyloside xylohydrolase
AL6G23510	none		DNA damage repair/tolerance DRT100-related (AT5G12940)^r^
AL8G32870	none	CPL1	cysteine protease-like 1^a^
AL8G32880	none		hypothetical protein AXX17-related (AT5G56200)^s^

^a^Large-effect SNPs or indels of allele frequency difference greater than 0.9 in at least one population pair.

^b^Tissue-specific or strongly enhanced gene expression: ^g^germination, ^s^siliques, ^r^root, ^f^flower, ^p^pollen, ^l^leaves.

### (f) Environmental association analysis

In a complementary approach taken to identify environmentally associated changes in primary protein structure, we conducted an EAA across all four sites, separately for each species. This analysis was justified by our analysis of population structure and by the differentiation in soil composition (see figure 1; electronic supplementary material, figure S4). We first identified candidate gene-coding loci positioned in a 25 SNP window exhibiting a signature of relative divergence (see Methods and the electronic supplementary material, Supplementary methods). For each gene identified in the DivergenceScan as overlapping with an outlier window, we subjected all SNPs of the coding region to an EAA using Bayenv2. Out of approximately 100 000 and 540 000 SNPs from transcribed regions tested for *A. halleri* and *A. arenosa* (see the electronic supplementary material, table S1), respectively, between 0.047% and 1.12% were identified to exhibit a strong environmental association (BF > 100, from Bayenv2). Among these, we additionally required that a candidate gene-coding locus must contain at least one associated non-synonymous SNP. We restricted this analysis to non-synonymous SNPs primarily because mapping coverage in intergenic regions in these highly heterozygous outcrossing plant species can be poor, whereas mapping to coding regions is very good. It is important to note that for both species we mapped sequencing reads against a diverged heterologous reference, *A. lyrata*. We further required that the environmentally associated non-reference allele must be present at a higher frequency in both M populations (Klet and Mias) compared to both NM populations. In other words, we required a change in primary protein structure to be derived. In *A. halleri*, a total of seven loci fulfilled all these stringent criteria, whereas for *A. arenosa*, no gene-coding locus was retained (table 1). None of the seven identified genes is associated with a pathway based on the NCBI biosystems repository. Thus, based on this stringent implementation of an EAA analysis, we did not find a single locus to be convergent between both species. This finding is consistent with the results of other less stringent approaches we applied with less success (not shown), such as the double outlier and Null-W test [50], a possible consequence of insufficient statistical power of our study including four populations and divergence metrics only.

### (g) Integrating results from both approaches

**Figure 4.**
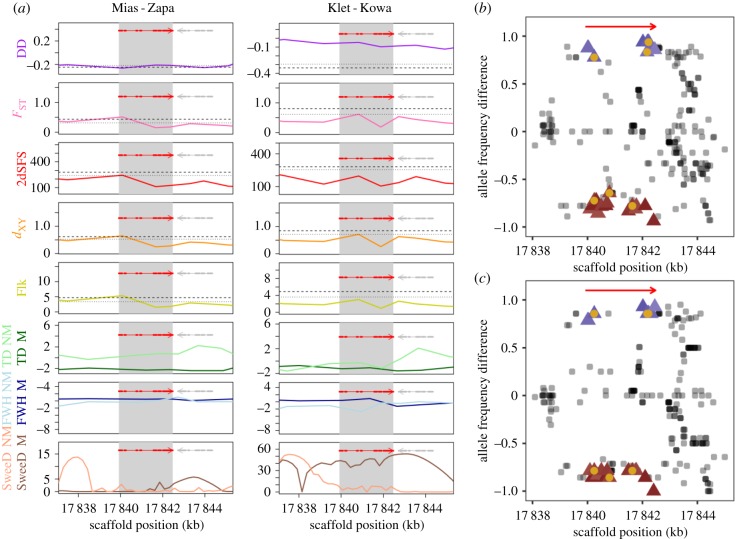
Evidence for convergent selection at the *A. halleri* candidate locus *Cysteine Protease-like 1* (*CPL1*, AL8G32870). (*a*) Genome scans. Lines connect datapoints, each representing the centre of a 25 SNP window, for diagnostic metrics for Mias versus Zapa (left) and Klet versus Kowa (right). The dashed/dotted lines mark the 99.9%/99.5% genome-wide percentiles (0.1%/0.5% for DD), respectively. Red thick/thin lines reflect exons/introns of candidates. (*b*,*c*) EAA results for *CPL1* (red arrow) for Mias-Zako (*b*) and Klet-Kowa (*c*). Each datapoint marks the position of one SNP and its allele frequency difference between the metallicolous (M) and non-metallicolous (NM) population. Blue/red triangles represent SNPs positively/negatively correlated and very strongly associated (BF ≥ 100) with exchangeable Cd concentrations in soil, with non-synonymous variants marked by yellow circles (identical results for exchangeable zinc concentrations; not shown). Note that the EAA was conducted across all four populations, whereas panels (*b*) and (*c*) show only pairwise allele frequency differences. More negative DD residuals (in *a*) indicate lowered diversity relative to the degree of between-population differentiation; the magnitude of *F*_ST_ values reflects the degree of between-population relative differentiation; raised 2dSFS values reflect a shift in the two-dimensional site frequency spectrum consistent with positive selection; *d_XY_* quantifies absolute net between-population absolute divergence; Flk indicates the degree of population differentiation adjusted for relatedness. A negative Tajima's *D* (TD) reflects an excess of low frequency variants; a negative Fay and Wu's H (FWH) an excess of high-frequency derived SNPs; elevated SweeD represents a shift in the site frequency spectrum indicating a selective sweep (see Methods). M (Mias, Klet)/NM populations are shown in dark/light colour in (*a*).

One candidate gene, *Cysteine Protease-like 1* (AL8G32870, *CPL1*), was identified in *A. halleri* by both (i) intersecting results of genome scans combined with potentially selected large-effect SNPs/indels (see §3d,e), and (ii) EAA of candidate genes in both M populations (see §3f) (table 1, electronic supplementary material, datasets S2 and S3, figure 4). The signature of selection over the candidate gene AL8G32870 was stronger for Mias-Zapa (≥99.9%ile for *F*_ST_, *d_XY_*, Flk, highly negative Tajima's *D*, ≤0.1% for DD) than for Klet-Kowa (figure 4*a*). *AhCPL1* was identified as a candidate gene in the Klet-Kowa population pair based on a large-effect indel exhibiting a high allele frequency difference (allele frequency difference = 0.86, electronic supplementary material, dataset S3). SweeD indicated a strong signal for the Klet (M2), but not the Kowa (NM2) population. Window-based divergence metrics gave *F*_ST_ and *d_XY_* values in the ≥99.5%ile and DD in the ≤0.5%ile in Klet-Kowa. Extreme values of metrics pinpointed exons one to four (figure 4*a*), in agreement with EAA which identified 50% of the significantly associated SNPs in this region (figure 4*b*,*c*). A closer inspection of haplotypes revealed that the predicted proteins at Mias (M1) and Klet (M2) share amino acid variants at 12 out of a total of 16 variable positions of the predicted protein that differentiate them from Zapa (NM1) and Kowa (NM2) (electronic supplementary material, figure S6). Of these 12 amino acids characteristic of M sites, nine are derived compared to the *A. lyrata* reference.

### (h) Homology modelling of *Ah*CPL1 proteins

To gain insight into possible consequences of the amino acid exchanges in the predicted *Ah*CPL1 Papain-like cysteine protease protein, we generated a homology model of the protein structure. An initial multiple sequence alignment (electronic supplementary material, figure S7*a*) suggested that a segment of the protein was missing (t2, *A. lyrata* genome v2.1, JGI Phytozome, https://phytozome.jgi.doe.gov/pz/portal.html). Intron–exon boundaries were then re-assessed based on *A. halleri* v1.1 (JGI Phytozome). We hypothetically expanded the 5′-end of the fifth exon by 177 nucleotides (59 amino acids), leading to the incorporation of the missing protein motifs in the metallicolous *Ah*CPL1 variants (electronic supplementary material, figures S7*b* and S8). In the structures generated by homology modelling the protein consists of an N-terminal 42-amino acid propeptide, which is likely to be cleaved during enzyme maturation. The mature protein consisted of characteristic L- and R-domains characterized by alpha helices and beta sheets, respectively (electronic supplementary material, figure S9, N-terminus included). While the protein is relatively divergent from other proteins with available cysteine protease structures, the active site is recognizable in the model and contains all four catalytically active residues in close proximity (Q62, C68, H212 and N234). However, with the intron–exon boundaries reconstructed here, a frameshift leading to a premature stop codon renders the predicted non-metallicolous *Ah*CPL1 variants non-functional.

## 4. Discussion

The objective of this study was to probe for candidate genes that have undergone convergent selection on calamine M soils. We focused on genes with evidence for selection at two M sites, each of them in comparison to a geographically proximal NM site, in the two closely related and genetically tractable species *A. halleri* and *A. arenosa* (electronic supplementary material, table S1). This sampling design was chosen in order to gain power [51] and test for convergence between species. We analysed genome-wide resequencing data obtained from field-collected individuals in relation to the mineral composition of root-proximal soils from the same individuals. Our objective of identifying convergent genes thus targeted selection by environmental factors common among (rather than specific to) population pairs.

The two M sites Mias and Klet were previously known for their vegetation type characteristic of calamine M soils [6]. In agreement with this, we found highly elevated Zn and Cd levels in root-proximal soils which distinguish both of these sites from the NM sites in this study (figure 1; electronic supplementary material, tables S2 and S3 and dataset S1). Our hypothesis of local adaptation to Mias soil in both species was supported by differing plant biomass production depending on population and soil type in independent experiments (figure 2; electronic supplementary material, table S4 and figures S2 and S3). In particular, upon cultivation on Mias M soil, plants of NM origin were more severely affected in *A. arenosa* than in *A. halleri*. Indeed, it is well-known that *A. halleri* exhibits enhanced hypertolerance to Zn and Cd species-wide [12,52], which is exceedingly rare [5,6]. Thus, local adaptation to M soil at Mias, and probably also at Klet, must confer a larger increment in heavy metal tolerance to *A. arenosa* than to *A. halleri*.

Genome scans identified few candidate genes being convergently under selection at both site pairs in either of the two species (figure 3). Similarly, the number of genes convergent between species across site pairs, i.e. between *A. arenosa* at Klet (versus Kowa) and *A. halleri* at Mias (versus Zapa) was very low, but both also exceeded the number expected by chance alone (electronic supplementary material, table S5). This latter finding was consistent with our expectations based on species-wide traits as outlined above, and with the fact that Zn and Cd concentrations were considerably lower in Klet soils than in Mias soils (electronic supplementary material, dataset S1).

At the level of GOs, protein localization was common to three out of four contrasts and is thus a candidate function under convergent selection (figure 3*b*). To date, functions in cellular protein trafficking are neither known as vulnerable targets of Zn or Cd toxicity nor as a means of attaining cellular metal tolerance. The cellular targets of heavy metal toxicity are very poorly understood to date.

Among the candidate genes exhibiting some degree of convergence, we identified genes known to act in the homeostasis of Fe (bHLH transcription factor-encoding *PYE* [53]) and Cu (cell surface Cu(II) chelate reductase-encoding *FRO4* [54]). Homeostasis of essential nutrients Fe and Cu is a well-known target of Zn and Cd toxicities [55–59] (table 1; electronic supplementary material, figure S5). Predicted functions of other convergent candidate genes were diverse, generally poorly characterized, and sometimes in a context of known relevance under heavy metal stress, for example cell wall composition or DNA damage repair [60,61]. These results suggest a modest degree of biologically relevant convergent evolution, more prominently within species and between sites, but also between species across environmental contrasts of comparable magnitude.

Based on existing knowledge of the molecular basis of species-wide metal hypertolerance, we had expected to identify candidate genes with direct roles in the detoxification of Cd^2+^ and Zn^2+^, such as their transmembrane transport and binding [11,21,62]. This was indeed the case in *A. arenosa* at one site pair (Mias-Zapa), yet with no apparent convergence (figure 3*b*). The genes underlying within-species variation in metal tolerance of plants are unknown. Almost all genes that have been experimentally demonstrated to contribute to naturally selected species-wide Zn and Cd hypertolerance, and also many candidate genes implicated in these traits, are copy number-expanded in *A. halleri*, with paralogues almost identical in sequence [62–64]. Genes with such sequence properties are usually disregarded in genome scans by excluding both reads mapping to multiple loci in the genome and genomic regions with excessive short read coverage. Beyond these technical issues, it can be very difficult to detect selective sweeps at such loci, because they commonly exhibit complex patterns of polymorphism resulting from ectopic gene conversion or illegitimate recombination. This was exemplified by the copy number-expanded metal hyperaccumulation and metal hypertolerance locus *HMA4* of *A. halleri* [65].

Through both genome scan and EAA approaches, we identified the *A. halleri* locus corresponding to AL8G32870 in the *A. lyrat*a reference genome as a convergent candidate gene between both population pairs (table 1 and figure 4; electronic supplementary material, datasets S6 and S7). In confirmation, we observed convergent high frequency non-synonymous sequence divergence at this locus specific to M sites (electronic supplementary material, figure S6). According to between-population differentiation metrics, this gene was thus not among the strongest selective sweep candidates in Klet-Kowa, but instead entered the list of candidates through an indel observed with a high between-population allele frequency difference (electronic supplementary material, dataset S3). *AhCPL1* is predicted to encode a papain family cysteine protease that has no orthologue in *A. thaliana* (electronic supplementary material, figure S7). An adjustment of the intron–exon boundaries raised the possibility that a functional CPL1 protein may be specific to the M sites Mias and Klet (electronic supplementary material, figure S8). We were not able to identify an *AhCPL1* cDNA encoding a functional protein using PCR on cDNA synthesized from total RNA extracted from leaves of individuals from the populations from this study, even with primers designed according to various alternative predictions. More extensive molecular approaches, also incorporating a comprehensive set of *A. halleri* organs, will be required to address the functional implications of the *AhCPL1* sequence variants identified here. *AhCPL1* is the first candidate metal hypertolerance gene of a Brassicaceae species lacking a homologue in a syntenic position in the *A. thaliana* genome. This finding may contribute to explaining why no *A. thaliana* accession was identified to grow naturally on M soil.

The possible molecular role of *Ah*CPL1 in metal tolerance remains unknown. The papain family cysteine protease genes of *A. thaliana Response to Dehydration 19* and *21* (*RD19*, *RD21*) have long been known as components of the transcriptional response to dehydration and salt stress [66]. More recently, RD19 was reported to function in signalling to trigger anti-microbial defences [67]. In wheat, a cysteine protease was transcriptionally upregulated under aluminium stress [68]. In *Chlamydomonas* sp., oxidative stress induced a cysteine protease which provided Cd tolerance [69]. In *A. halleri*, transcript levels encoding a putative cysteine proteinase (AT2G27420) were observed to be about 30-fold higher than in *A. thaliana* according to microarray-based cross-species transcriptomics [70].

It must be kept in mind that there were several important environmental factors differing between sites. Specifically, when compared to all other soils, Mias M soil was between 3- and 10-fold lower in exchangeable Ca^2+^, a nutritional condition that is well known to enhance the toxicity of divalent heavy metal cations [71]. Mias soil was also lower in several nutrients, i.e. exchangeable K^+^, Mg^2+^ and Mn^2+^, in contrast to the Klet-Kowa pair (electronic supplementary material, dataset S1 and table S2). Importantly, Mias soils were on average more than 100-fold higher in exchangeable Pb than Zapa NM soils. Pb is a heavy metal with extremely high toxicity potential. By contrast, soil exchangeable Pb was not elevated at Klet (versus Kowa). Conversely, Klet soils contained about fourfold elevated exchangeable concentrations of Cu, which can be highly toxic to plants. As a former uranium mine, Klet soil may additionally contain elevated levels of toxic decay products of uranium that were not quantified here (e.g. polonium, thallium). Consequently, the limited number of convergent candidate genes identified here may relate to the multi-factorial stress of local soil environments (figure 3; electronic supplementary material, datasets S2–S7). Future experiments should now address the precise degree of phenotypic convergence by testing for local soil adaptation in the Klet-Kowa pair in both species and by testing the performance of plants from the Klet M site on Mias M soil, and vice versa.

The candidates obtained through the sequence divergence-based approaches pursued in this study, and their intersection, overall suggested a limited sensitivity of these approaches. Nevertheless, we could identify several candidate genes convergent between site pairs and between species, as well as convergent sequence variants in one convergent candidate gene. Our data suggest the existence of functional gene network convergence, but with partially differing proximal molecular factors mediating functional convergence. Considerable future effort will be required for the functional characterization of identified candidate genes and networks. Additionally, a greater degree of convergent evolution within species may be observed in future work at M sites chosen for higher similarity in soil composition, whereas here we chose M sites exclusively based on the presence of both species.

## Supplementary Material

Figure S1

## Supplementary Material

Figure S2

## Supplementary Material

Figure S3

## Supplementary Material

Figure S4

## Supplementary Material

Figure S5

## Supplementary Material

Figure S6

## Supplementary Material

Figure S7

## Supplementary Material

Figure S8

## Supplementary Material

Figure S9

## Supplementary Material

Supplementary methods

## Supplementary Material

Table S1

## Supplementary Material

Dataset S1

## Supplementary Material

Dataset S2

## Supplementary Material

Dataset S3

## Supplementary Material

Dataset S4

## Supplementary Material

Dataset S5

## Supplementary Material

Dataset S6

## Supplementary Material

Dataset S7

## Data accessibility

Sequence data that support the findings of this study have been deposited in the Sequence Read Archive (SRA; https://www.ncbi.nlm.nih.gov/sra) with the primary accession code PRJNA506705 (available at http://www.ncbi.nlm.nih.gov/bioproject/506705). Genome-wide analysis results are available from the Dryad Digital Repository: https://doi.org/10.5061/dryad.jg30j4v [72].

## References

[1] Nieboer E, Richardson DHS 1980 The replacement of the nondescript term ‘heavy metals’ by a biologically and chemically significant classification of metal ions. Environ. Pollut. B – Chem. Phys. 1, 3–26. (10.1016/0143-148X(80)90017-8)

[2] Brady KU, Kruckeberg AR, Bradshaw HDJ 2005 Evolutionary ecology of plant adaptation to serpentine soils. Annu. Rev. Ecol. Evol. Syst. 36, 243–266. (10.1146/annurev.ecolsys.35.021103.105730)

[3] Wójcik M, Gonnelli C, Selvi F, Dresler S, Rostański A, Vangronsveld J 2017 Chapter One—Metallophytes of serpentine and calamine soils—their unique ecophysiology and potential for phytoremediation. In Advances in botanical research (eds Cuypers A, Vangronsveld J), pp. 1–42. London, UK: Academic Press.

[4] Ernst WHO 1974 Schwermetallvegetationen der Erde. Stuttgart, Germany: Gustav Fischer Verlag.

[5] Bert V, MacNair MR, De Laguérie P, Saumitou-Laprade P, Petit D. 2000 Zinc tolerance and accumulation in metallicolous and non metallicolous populations of *Arabidopsis halleri* (Brassicaceae). New Phytol. 146, 225–233. (10.1046/j.1469-8137.2000.00634.x) 33862970

[6] Stein RJ, Höreth S, de Melo JR, Syllwasschy L, Lee G, Garbin ML, Clemens S, Krämer U. 2017 Relationships between soil and leaf mineral composition are element-specific, environment-dependent and geographically structured in the emerging model *Arabidopsis halleri*. New Phytol. 213, 1274–1286. (10.1111/nph.14219) 27735064 PMC5248639

[7] Przedpelska E, Wierzbicka M 2007 *Arabidopsis arenosa* (Brassicaceae) from a lead-zinc mine waste heap in southern Poland. Plant Soil 299, 43–53. (10.1007/s11104-007-9359-5)

[8] Turisova I, Strba T, Aschenbrenner S, Andras P 2013 *Arabidopsis arenosa* (L.) Law. On metalliferous and non-metalliferous sites in central Slovakia. Bull. Environ. Contam. Toxicol. 91, 469–474. (10.1007/s00128-013-1074-8) 23912231

[9] Arnold BJ, Lahner B, DaCosta JM, Weisman CM, Hollister JD, Salt DE, Bomblies K, Yant L 2016 Borrowed alleles and convergence in serpentine adaptation. Proc. Natl Acad. Sci. USA 113, 8320–8325. (10.1073/pnas.1600405113) 27357660 PMC4961121

[10] Turner TL, Bourne EC, Von Wettberg EJ, Hu TT, Nuzhdin SV. 2010 Population resequencing reveals local adaptation of *Arabidopsis lyrata* to serpentine soils. Nat. Genet. 42, 260–263. (10.1038/ng.515) 20101244

[11] Krämer U 2010 Metal hyperaccumulation in plants. Annu. Rev. Plant Biol. 61, 517–534. (10.1146/annurev-arplant-042809-112156) 20192749

[12] Meyer CL, Juraniec M, Huguet S, Chaves-Rodriguez E, Salis P, Isaure MP, Goormaghtigh E, Verbruggen N 2015 Intraspecific variability of cadmium tolerance and accumulation, and cadmium-induced cell wall modifications in the metal hyperaccumulator *Arabidopsis halleri*. J. Exp. Bot. 66, 3215–3227. (10.1093/jxb/erv144) 25873677 PMC4449548

[13] Meyer CL, Kostecka AA, Saumitou-Laprade P, Creach A, Castric V, Pauwels M, Frerot H 2010 Variability of zinc tolerance among and within populations of the pseudometallophyte species *Arabidopsis halleri* and possible role of directional selection. New Phytol. 185, 130–142. (10.1111/j.1469-8137.2009.03062.x) 19863732

[14] Clemens S 2001 Molecular mechanisms of plant metal tolerance and homeostasis. Planta 212, 475–486. (10.1007/s004250000458) 11525504

[15] Peer WA, Mahmoudian M, Freeman JL, Lahner B, Richards EL, Reeves RD, Murphy AS, Salt DE 2006 Assessment of plants from the Brassicaceae family as genetic models for the study of nickel and zinc hyperaccumulation. New Phytol. 172, 248–260. (10.1111/j.1469-8137.2006.01820.x) 16995913

[16] Szarek-Łukaszewska G, Niklińska M 2002 Concentration of alkaline and heavy metals in *Biscutella laevigata* L. and *Plantago lanceolata* L. growing on calamine spoils (S. Poland). Acta Biol. Crac. Ser. Bot. 44, 29–38.

[17] Szarek-Łukaszewska G, Grodzińska K 2011 Grasslands of a Zn-Pb post-mining area (Olkusz ore-bearing region, S Poland). Pol. Bot. J. 56, 245–260.

[18] Kenderesova L, Stanova A, Pavlovkin J, Durisova E, Nadubinska M, Ciamporova M, Ovecka M 2012 Early Zn^2+^-induced effects on membrane potential account for primary heavy metal susceptibility in tolerant and sensitive *Arabidopsis* species. Ann. Bot. 110, 445–459. (10.1093/aob/mcs111) 22645116 PMC3394654

[19] Nadgorska-Socha A, Ptasinski B, Kita A 2013 Heavy metal bioaccumulation and antioxidative responses in *Cardaminopsis arenosa* and *Plantago lanceolata* leaves from metalliferous and non-metalliferous sites: a field study. Ecotoxicology 22, 1422–1434. (10.1007/s10646-013-1129-y) 24085602 PMC3824952

[20] Hanikenne M, Nouet C 2011 Metal hyperaccumulation and hypertolerance: a model for plant evolutionary genomics. Curr. Opin. Plant Biol. 14, 252–259. (10.1016/j.pbi.2011.04.003) 21531166

[21] Verbruggen N, Hermans C, Schat H 2009 Molecular mechanisms of metal hyperaccumulation in plants. New Phytol. 181, 759–776. (10.1111/j.1469-8137.2008.02748.x) 19192189

[22] Douglas B, Martin M, Ben B, Steve W 2015 Fitting linear mixed-effects models using lme4. J. Stat. Softw. 67, 1–48. (10.18637/jss.v067.i01)

[23] John F, Sanford W, 2011 An {R} companion to applied regression, 2nd edn Thousand Oaks, CA: Sage.

[24] Rogstad SH 1992 Saturated NaCl-CTAB solution as a means of field preservation of leaves for DNA analyses. Taxon 41, 701–708. (10.2307/1222395)

[25] McKenna A et al 2010 The genome analysis toolkit: a MapReduce framework for analyzing next-generation DNA sequencing data. Genome Res. 20, 1297–1303. (10.1101/gr.107524.110) 20644199 PMC2928508

[26] Lê S, Josse J, Husson F 2008 FactoMineR: an R package for multivariate analysis. J. Stat. Softw. 25, 18 (10.18637/jss.v025.i01)

[27] Yant L, Hollister JD, Wright KM, Arnold BJ, Higgins JD, Franklin FC. H, Bomblies K 2013 Meiotic adaptation to genome duplication in *Arabidopsis arenosa*. Curr. Biol. 23, 2151–2156. (10.1016/j.cub.2013.08.059) 24139735 PMC3859316

[28] Ross-Ibarra J, Wright SI, Foxe JP, Kawabe A, DeRose-Wilson L, Gos G, Charlesworth D, Gaut BS 2008 Patterns of polymorphism and demographic history in natural populations of *Arabidopsis lyrata*. PLoS ONE 3, e2411 (10.1371/journal.pone.0002411) 18545707 PMC2408968

[29] Wright S 1951 The genetical structure of populations. Ann. Eugen. 15, 323–354. (10.1111/j.1469-1809.1949.tb02451.x) 24540312

[30] Nielsen R et al. 2009 Darwinian and demographic forces affecting human protein coding genes. Genome Res. 19, 838–849. (10.1101/gr.088336.108) 19279335 PMC2675972

[31] Smith J, Kronforst MR 2013 Do *Heliconius* butterfly species exchange mimicry alleles? Biol. Lett. 9, 20130503 (10.1098/rsbl.2013.0503) 23864282 PMC3730661

[32] Bonhomme M, Chevalet C, Servin B, Boitard S, Abdallah J, Blott S, SanCristobal M 2010 Detecting selection in population trees: the Lewontin and Krakauer test extended. Genetics 186, 241–262. (10.1534/genetics.110.117275) 20855576 PMC2940290

[33] Teo YY, Fry AE, Bhattacharya K, Small KS, Kwiatkowski DP, Clark TG 2009 Genome-wide comparisons of variation in linkage disequilibrium. Genome Res. 19, 1849–1860. (10.1101/gr.092189.109) 19541915 PMC2765270

[34] Tajima F 1989 Statistical method for testing the neutral mutation hypothesis by DNA polymorphism. Genetics 123, 585–595. 2513255 10.1093/genetics/123.3.585 PMC1203831

[35] Fay JC, Wu C-I 2000 Hitchhiking under positive Darwinian selection. Genetics 155, 1405–1413. 10880498 10.1093/genetics/155.3.1405 PMC1461156

[36] Pavlidis P, Zivkovic D, Stamatakis A, Alachiotis N 2013 SweeD: likelihood-based detection of selective sweeps in thousands of genomes. Mol. Biol. Evol. 30, 2224–2234. (10.1093/molbev/mst112) 23777627 PMC3748355

[37] Cruickshank TE, Hahn MW 2014 Reanalysis suggests that genomic islands of speciation are due to reduced diversity, not reduced gene flow. Mol. Ecol. 23, 3133–3157. (10.1111/mec.12796) 24845075

[38] Oleksyk TK, Smith MW, O'Brien SJ 2010 Genome-wide scans for footprints of natural selection. Phil. Trans. R. Soc. B 365, 185–205. (10.1098/rstb.2009.0219) 20008396 PMC2842710

[39] Cingolani P, Platts A, Wang L, Coon M, Nguyen T, Wang L, Land SJ, Lu X, Ruden DM 2012 A program for annotating and predicting the effects of single nucleotide polymorphisms, SnpEff: SNPs in the genome of *Drosophila melanogaster* strain w1118; iso-2; iso-3. Fly (Austin) 6, 80–92. (10.4161/fly.19695) 22728672 PMC3679285

[40] Rawat V, Abdelsamad A, Pietzenuk B, Seymour DK, Koenig D, Weigel D, Pecinka A, Schneeberger K 2015 Improving the annotation of *Arabidopsis lyrata* using RNA-Seq data. PLoS ONE 10, e0137391 (10.1371/journal.pone.0137391) 26382944 PMC4575116

[41] Oliveros JC 2007–2015 Venny. An interactive tool for comparing lists with Venn's diagrams. See http://bioinfogp.cnb.csic.es/tools/venny/index.html.

[42] Bindea G et al. 2009 ClueGO: a Cytoscape plug-in to decipher functionally grouped gene ontology and pathway annotation networks. Bioinformatics 25, 1091–1093. (10.1093/bioinformatics/btp101) 19237447 PMC2666812

[43] Shannon P, Markiel A, Ozier O, Baliga NS, Wang JT, Ramage D, Amin N, Schwikowski B, Ideker T 2003 Cytoscape: a software environment for integrated models of biomolecular interaction networks. Genome Res. 13, 2498–2504. (10.1101/gr.1239303) 14597658 PMC403769

[44] Gunther T, Coop G 2013 Robust identification of local adaptation from allele frequencies. Genetics 195, 205–220. (10.1534/genetics.113.152462) 23821598 PMC3761302

[45] Jeffreys H 1961 The theory of probability, 3rd edn Oxford, UK: Oxford University Press.

[46] Šali A, Blundell TL 1993 Comparative protein modelling by satisfaction of spatial restraints. J. Mol. Biol. 234, 779–815. (10.1006/jmbi.1993.1626) 8254673

[47] Buchan DW. A., Minneci F, Nugent TC. O., Bryson K, Jones DT 2013 Scalable web services for the PSIPRED protein analysis workbench. Nucleic Acids Res. 41, W349–W357. (10.1093/nar/gkt381) 23748958 PMC3692098

[48] Waterhouse AM, Procter JB, Martin DM. A., Clamp M, Barton GJ 2009 Jalview Version 2—a multiple sequence alignment editor and analysis workbench. Bioinformatics 25, 1189–1191. (10.1093/bioinformatics/btp033) 19151095 PMC2672624

[49] Briskine RV, Paape T, Shimizu-Inatsugi R, Nishiyama T, Akama S, Sese J, Shimizu KK 2017 Genome assembly and annotation of *Arabidopsis halleri*, a model for heavy metal hyperaccumulation and evolutionary ecology. Mol. Ecol. Resour. 17, 1025–1036. (10.1111/1755-0998.12604) 27671113

[50] Yeaman S et al 2016 Convergent local adaptation to climate in distantly related conifers. Science 353, 1431–1433. (10.1126/science.aaf7812) 27708038

[51] Lotterhos KE, Whitlock MC 2015 The relative power of genome scans to detect local adaptation depends on sampling design and statistical method. Mol. Ecol. 24, 1031–1046. (10.1111/mec.13100) 25648189

[52] Pauwels M, Frerot H, Bonnin I, Saumitou-Laprade P 2006 A broad-scale analysis of population differentiation for Zn tolerance in an emerging model species for tolerance study: *Arabidopsis halleri* (Brassicaceae). J. Evol. Biol. 19, 1838–1850. (10.1111/j.1420-9101.2006.01178.x) 17040381

[53] Long TA, Tsukagoshi H, Busch W, Lahner B, Salt DE, Benfey PN 2010 The bHLH transcription factor POPEYE regulates response to iron deficiency in *Arabidopsis* roots. Plant Cell 22, 2219–2236. (10.1105/tpc.110.074096) 20675571 PMC2929094

[54] Bernal M et al 2012 Transcriptome sequencing identifies SPL7-regulated copper acquisition genes FRO4/FRO5 and the copper dependence of iron homeostasis in *Arabidopsis*. Plant Cell 24, 738–761. (10.1105/tpc.111.090431) 22374396 PMC3315244

[55] Gayomba SR et al 2013 The CTR/COPT-dependent copper uptake and SPL7-dependent copper deficiency responses are required for basal cadmium tolerance in *A. thaliana*. Metallomics 5, 1262–1275. (10.1039/c3mt00111c) 23835944

[56] Connolly EL, Fett JP, Guerinot ML 2002 Expression of the IRT1 metal transporter is controlled by metals at the levels of transcript and protein accumulation. Plant Cell 14, 1347–1357. (10.1105/tpc.001263) 12084831 PMC150784

[57] Vert G, Grotz N, Dedaldechamp F, Gaymard F, Guerinot ML, Briat JF, Curie C 2002 IRT1, an *Arabidopsis* transporter essential for iron uptake from the soil and for plant growth. Plant Cell 14, 1223–1233. (10.1105/tpc.001388) 12084823 PMC150776

[58] Arrivault S, Senger T, Krämer U 2006 The *Arabidopsis* metal tolerance protein AtMTP3 maintains metal homeostasis by mediating Zn exclusion from the shoot under Fe deficiency and Zn oversupply. Plant J. 46, 861–879. (10.1111/j.1365-313X.2006.02746.x) 16709200

[59] Connolly EL, Campbell NH, Grotz N, Prichard CL, Guerinot ML 2003 Overexpression of the FRO2 ferric chelate reductase confers tolerance to growth on low iron and uncovers posttranscriptional control. Plant Physiol. 133, 1102–1110. (10.1104/pp.103.025122) 14526117 PMC281606

[60] Cosio C, DeSantis L, Frey B, Diallo S, Keller C 2005 Distribution of cadmium in leaves of *Thlaspi caerulescens*. J. Exp. Bot. 56, 765–775. (10.1093/jxb/eri062) 15642714

[61] Kovalchuk O, Titov V, Hohn B, Kovalchuk I 2001 A sensitive transgenic plant system to detect toxic inorganic compounds in the environment. Nat. Biotechnol. 19, 568–572. (10.1038/89327) 11385463

[62] Hanikenne M, Talke IN, Haydon MJ, Lanz C, Nolte A, Motte P, Kroymann J, Weigel D, Krämer U 2008 Evolution of metal hyperaccumulation required *cis*-regulatory changes and triplication of *HMA4*. Nature 453, 391–395. (10.1016/0143-148X(80)90017-8) 18425111

[63] Dräger DB, Desbrosses-Fonrouge AG, Krach C, Chardonnens AN, Meyer RC, Saumitou-Laprade P, Krämer U 2004 Two genes encoding *Arabidopsis halleri* MTP1 metal transport proteins co-segregate with zinc tolerance and account for high MTP1 transcript levels. Plant J. 39, 425–439. (10.1111/j.1365-313X.2004.02143.x) 15255871

[64] Suryawanshi V, Talke IN, Weber M, Eils R, Brors B, Clemens S, Kramer U 2016 Between-species differences in gene copy number are enriched among functions critical for adaptive evolution in *Arabidopsis halleri*. BMC Genomics 17, 1034 (10.1186/s12864-016-3319-5) 28155655 PMC5259951

[65] Hanikenne M, Kroymann J, Trampczynska A, Bernal M, Motte P, Clemens S, Krämer U 2013 Hard selective sweep and ectopic gene conversion in a gene cluster affording environmental adaptation. PLoS Genet. 9, e1003707 (10.1371/journal.pgen.1003707) 23990800 PMC3749932

[66] Koizumi M, Yamaguchi-Shinozaki K, Tsuji H, Shinozaki K 1993 Structure and expression of two genes that encode distinct drought-inducible cysteine proteinases in *Arabidopsis thaliana*. Gene 129, 175–182. (10.1016/0378-1119(93)90266-6) 8325504

[67] Bernoux M, Timmers T, Jauneau A, Briere C, de Wit PJ, Marco Y, Deslandes L. 2008 RD19, an *Arabidopsis* cysteine protease required for RRS1-R-mediated resistance, is relocalized to the nucleus by the *Ralstonia solanacearum* PopP2 effector. Plant Cell 20, 2252–2264. (10.1105/tpc.108.058685) 18708476 PMC2553607

[68] Hamel F, Breton C, Houde M 1998 Isolation and characterization of wheat aluminum-regulated genes: possible involvement of aluminum as a pathogenesis response elicitor. Planta 205, 531–538. (10.1007/s004250050352) 9684357

[69] Usui M, Tanaka S, Miyasaka H, Suzuki Y, Shioi Y 2007 Characterization of cysteine protease induced by oxidative stress in cells of *Chlamydomonas* sp. strain W80. Physiol. Plant. 131, 519–526. (10.1111/j.1399-3054.2007.00981.x) 18251844

[70] Becher M, Talke IN, Krall L, Krämer U 2004 Cross-species microarray transcript profiling reveals high constitutive expression of metal homeostasis genes in shoots of the zinc hyperaccumulator *Arabidopsis halleri*. Plant J. 37, 251–268. (10.1046/j.1365-313X.2003.01959.x) 14690509

[71] Woolhouse HW 1983 Toxicity and tolerance in the response of plants to metals. In Encyclopedia of plant physiology, New series (eds Lange OL, Nobel PS, Osmond CB, Ziegler H), pp. 245–300. Berlin, Germany: Springer.

[72] Preite V, Sailer C, Syllwasschy L, Bray S, Ahmadi H, Krämer U, Yant L 2019 Data from: Convergent evolution in *Arabidopsis halleri* and *Arabidopsis arenosa* on calamine metalliferous soils Dryad Digital Repository. (10.5061/dryad.jg30j4v) PMC6560266 31154972

