# Supplementary material for: Convergent evolution in Arabidopsis halleri and Arabidopsis arenosa on calamine metalliferous soils
Source: Philos Trans R Soc Lond B Biol Sci. 2019 Jun 3;374(1777):20180243. doi: 10.1098/rstb.2018.0243 (PMC6560266; doi:10.1098/rstb.2018.0243)
Supplement: Figure S1 [file rstb20180243supp1.pdf]

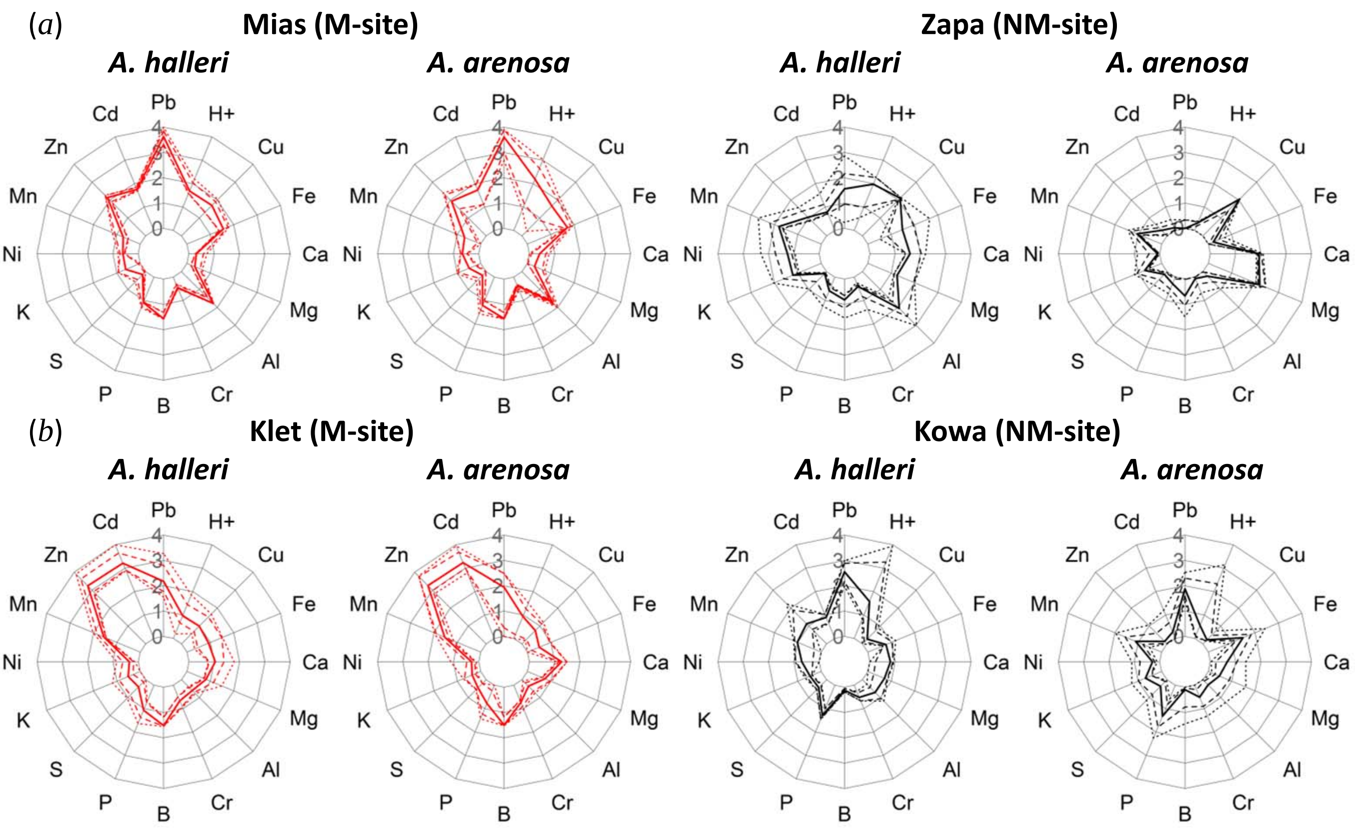

Figure S1. Mineral composition of extractable fraction of soils, and soil pH. (*a*) Site pair of Miasteczko Śląskie (Mias) and Zakopane (Zapa), (*b*) Site pair of Kletno (Klet) and Kowary (Kowa). The concentrations of multiple elements were determined in 0.1 M HCl extracts of soils collected directly adjacent to roots of the plant individuals sampled for this study (see Methods). Concentrations [mg element kg^{-1} dry soil mass] were normalized to the global minimum per site pair across both species, and subsequently Log_{10}-transformed. Shown are the median (solid line), 10 and 90%iles (dashed lines), minimum and maximum (dotted lines) for each site per species (n = 5 to 9 plant individuals) for metalliferous (M, red colour) and non-metalliferous (NM, black colour) soil types. Soil pH is re-plotted here from figure 1.
