# Supplementary material for: Convergent evolution in Arabidopsis halleri and Arabidopsis arenosa on calamine metalliferous soils
Source: Philos Trans R Soc Lond B Biol Sci. 2019 Jun 3;374(1777):20180243. doi: 10.1098/rstb.2018.0243 (PMC6560266; doi:10.1098/rstb.2018.0243)
Supplement: Figure S2 [file rstb20180243supp2.pdf]

(a)

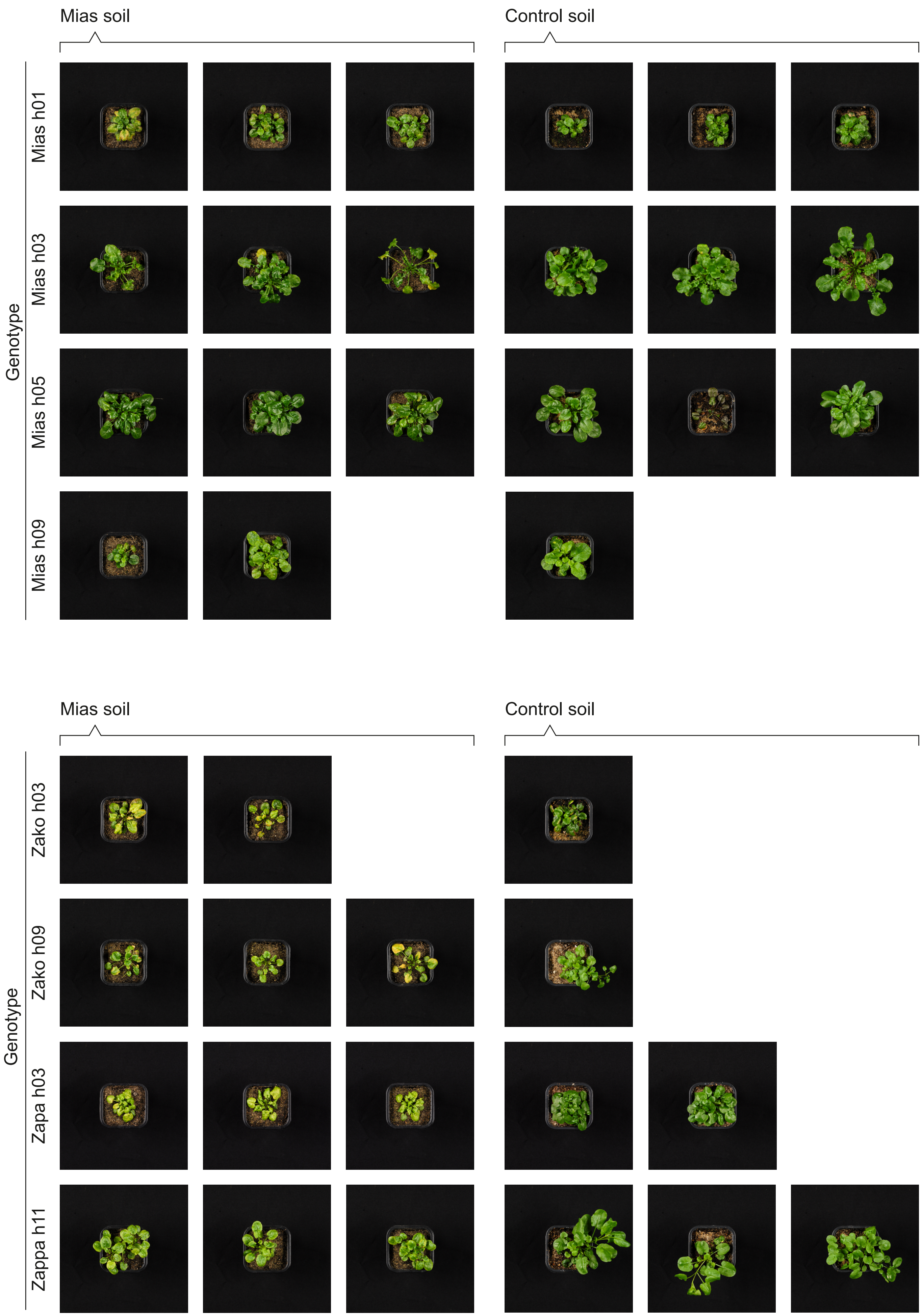
Mias soil
Control soil
Genotype
Mias h01
Mias h03
Mias h05
Mias h09
Mias soil
Control soil
Genotype
Zako h03
Zako h09
Zapa h03
Zappa h11

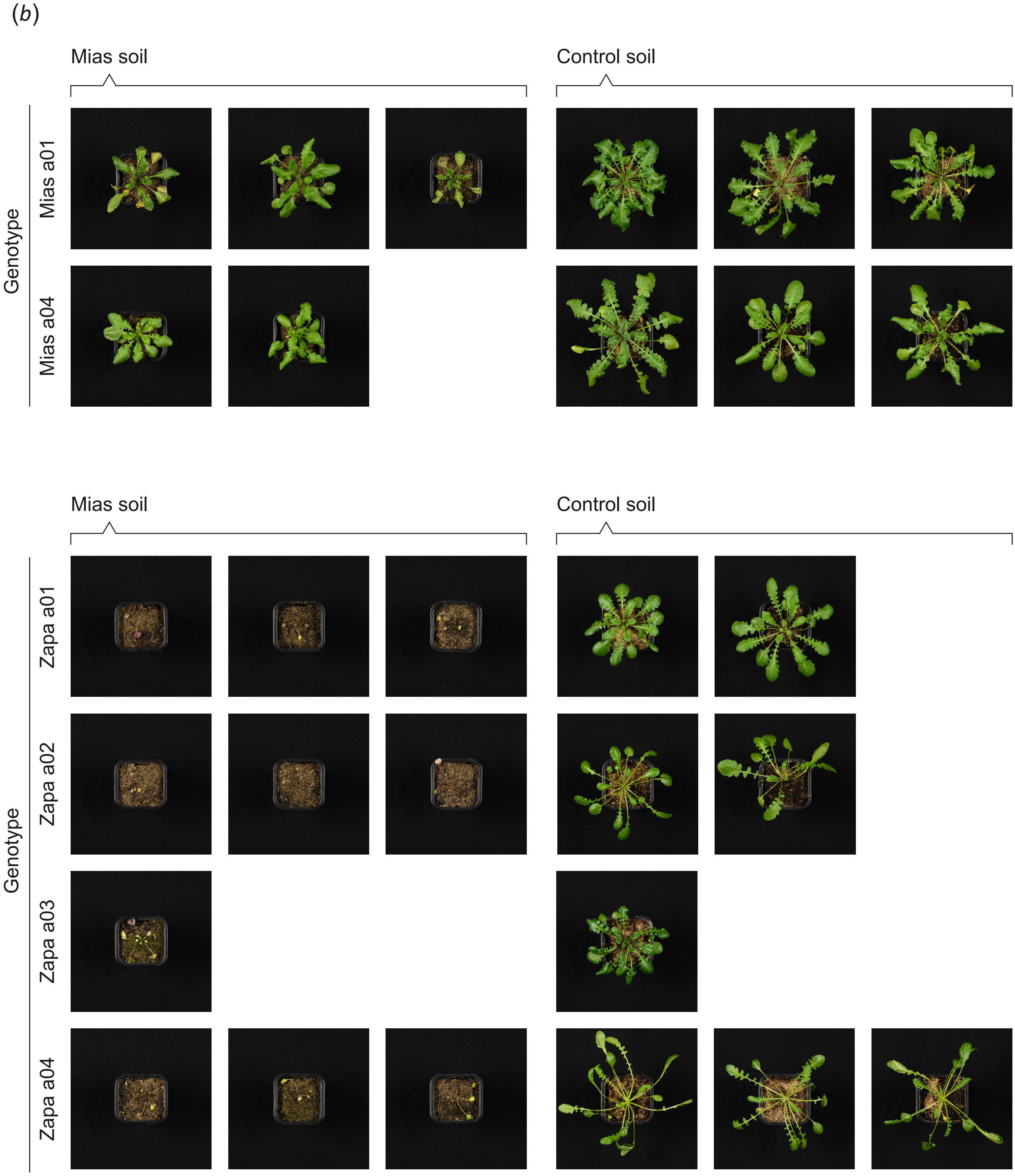

Figure S2. Photographs of plants in experiment testing for plant adaptation to metalliferous soil. (*a*) *A. halleri*, (*b*) *A. arenosa*. Photographs were taken of plants on the day of harvest (see figure 2).
