# Supplementary material for: Convergent evolution in Arabidopsis halleri and Arabidopsis arenosa on calamine metalliferous soils
Source: Philos Trans R Soc Lond B Biol Sci. 2019 Jun 3;374(1777):20180243. doi: 10.1098/rstb.2018.0243 (PMC6560266; doi:10.1098/rstb.2018.0243)
Supplement: Figure S3 [file rstb20180243supp3.pdf]

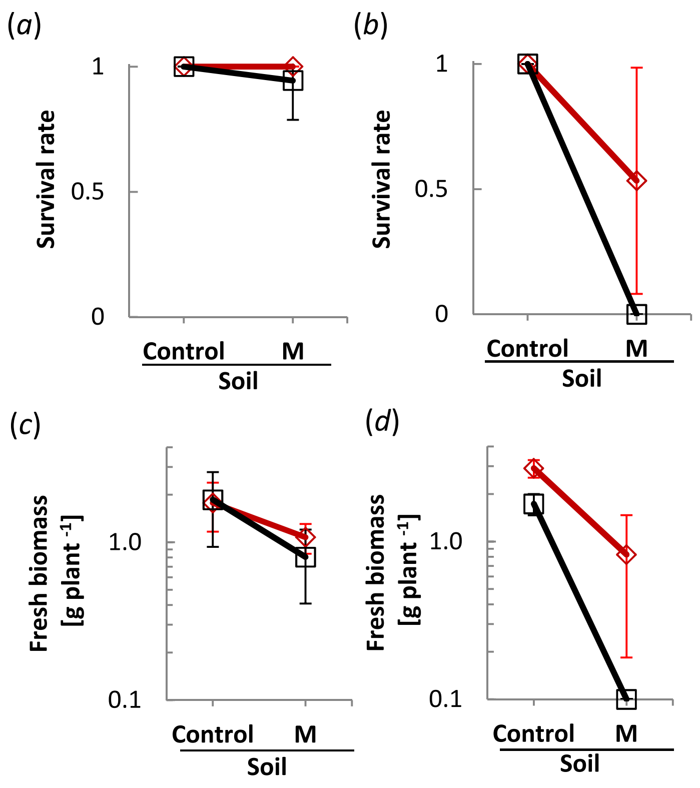

Figure S3. Independent experiment addressing local adaptation to metalliferous soil. (*a*,*b*) Survival of *A. halleri* (*a*) and *A. arenosa* (*b*) plants originating from Mias (M site, red colour) and Zapa (NM site, black colour) transferred into metalliferous (Mias) or non-metalliferous (control) soil. (c,*d*) Fresh biomass of *A. halleri* (*c*) and *A. arenosa* (*d*) plants originating from Mias (M site, red colour) and Zapa (NM site, black colour) transferred into metalliferous (Mias) or non-metalliferous (control) soil. Shown are means and standard deviation of survival rate (*a*,*b*) and fresh above-ground biomass (*c*,*d*) after 6 w of cultivation on experimental soils (see table S4 for details).
