# Supplementary material for: Convergent evolution in Arabidopsis halleri and Arabidopsis arenosa on calamine metalliferous soils
Source: Philos Trans R Soc Lond B Biol Sci. 2019 Jun 3;374(1777):20180243. doi: 10.1098/rstb.2018.0243 (PMC6560266; doi:10.1098/rstb.2018.0243)
Supplement: Figure S4 [file rstb20180243supp4.pdf]

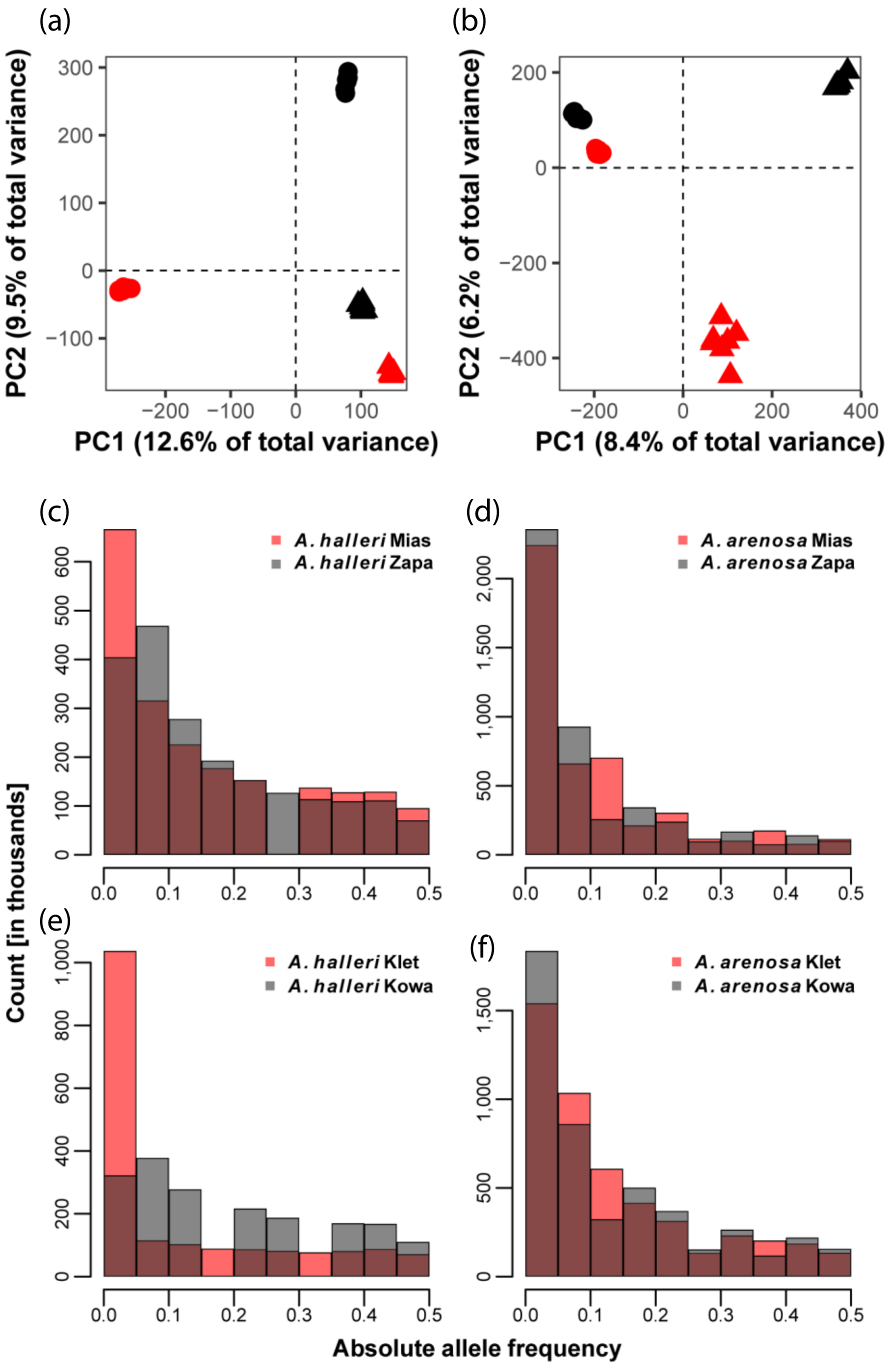

Figure S4. Population structure. (a, b) Principal component analysis of neutral population structure in A. halleri (a) and A. arenosa (b), based on putatively neutral four-fold degenerate sites (329,044 SNPs and 34 individuals for A. halleri, 699,168 SNPs and 31 individuals for A. arenosa). Each datapoint represents one plant individual sampled at a metalliferous (red symbols) or a non-metalliferous (black symbols) site of the Mias-Zapa (triangles) or Klet-Kowa (circles) population pair. (c-f) Folded site frequency spectra based on all SNPs at Mias-Zapa in A. halleri (c) and A. arenosa (d), and Klet-Kowa in A. halleri (e) and A. arenosa (f). Metalliferous sites are shown in red, non-metalliferous sites in grey.
