# Supplementary material for: Convergent evolution in Arabidopsis halleri and Arabidopsis arenosa on calamine metalliferous soils
Source: Philos Trans R Soc Lond B Biol Sci. 2019 Jun 3;374(1777):20180243. doi: 10.1098/rstb.2018.0243 (PMC6560266; doi:10.1098/rstb.2018.0243)
Supplement: Figure S5 [file rstb20180243supp5.pdf]

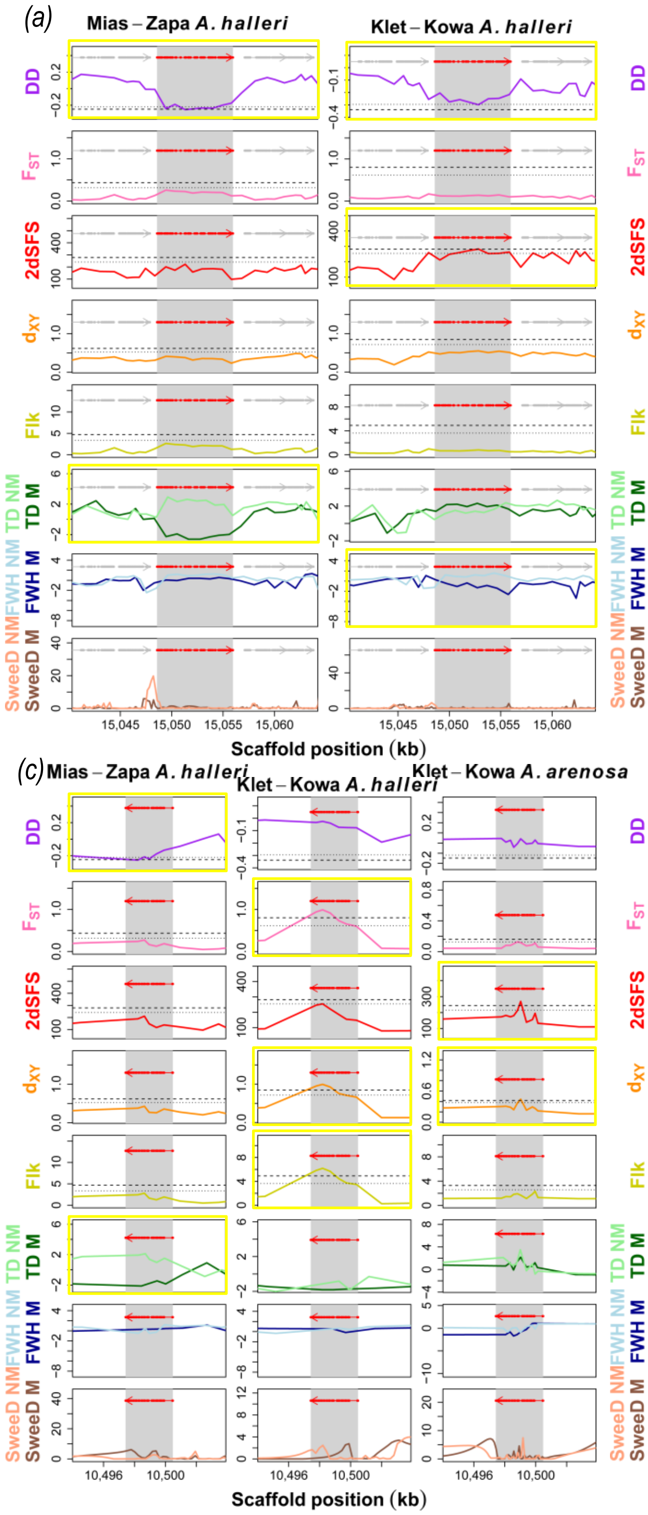

Figure S5. Evidence for selection on convergent genes. Lines connect datapoints, each representing the centre of a 25-SNP window, for a series of metrics for the respective population pairs for *(a)* AI2g31400, *(b)* AI7g35050, *(c)* AI8g20240, *(d)* AI6g35310. The dashed/dotted lines mark the 0.1%/0.5% genome-wide outliers. Thick/thin lines reflect exons/introns of candidate (red arrow, grey background) and neighbouring genes.
