# Supplementary material for: Convergent evolution in Arabidopsis halleri and Arabidopsis arenosa on calamine metalliferous soils
Source: Philos Trans R Soc Lond B Biol Sci. 2019 Jun 3;374(1777):20180243. doi: 10.1098/rstb.2018.0243 (PMC6560266; doi:10.1098/rstb.2018.0243)
Supplement: Figure S6 [file rstb20180243supp6.pdf]

```
Exon number     2222222222 2222222222 2233333333 3333333333 3344444444 4444444444 4444455555 5555555555
                ....|....| ....|....| ....|....| ....|....| ....|....| ....|....| ....|....| ....|....|
                    5         15         25         35         45         55         65         75
A. lyrata t2    MFTRSNRKGG GGNGEEKKNR FTAEEKGKGK MPSKSSKEEL WELPPSWDWR DYPGVIGPVM NQKLQGFCTA SQCTPNTRDN
Klet            MFTRSNRKGG GGNGEERKNR FTAEEKGKEK MPSKSSKEES SELPPSWDWR DYPGVIGPVM NQKLQGFCTA SQCTPNTRDN
Kowa            MFTRSNRKGG GGNGEENKNR FTAEEKGKGK MPSKSSKEES AELPPSWDWR DYPGVIGPVM NQKLEGFCTA SQCTPNTRDN
Mias            MFTRSNRKGG GGNGEERKNR FTAEEKGKEK MPSKSSKEES SELPPSWDWR DYPGVIGPVM NQKLQGFCTA SQCTPNTRDN
Zapa            MFTRSNRKGG GGNGEENKNR FTAEEKGKGK MPSKSSKEES AELPPSWDWR DYPGVIGPVM NQKLEGFCTA SQCTPNTRDN

Exon number     5555555555 5556666666 6666666666 6666666666 6666666666 6666666667 7777777777 7777777777
                ....|....| ....|....| ....|....| ....|....| ....|....| ....|....| ....|....| ....|....|
                     85         95        105        115        125        135        145        155
A. lyrata t2    NVFKKLVCRH PDNIHY-IKV DEFEYLTNVN DEELQAIVVQ QPVIGILRNT NDEFLAIGSG IYRSPSGDVD VNFHQVLIIG
Klet            NVFKKLVCRH PDNIHYNIKV DEFEYLTNVN DEELQAIVVQ QPVIGILRNT NDDFLGIGSG IYRSPSGDVD INFHQVLIIG
Kowa            NVFKKLVCRH PDKIHT-IKV DEFEYLTNVN DEELQAIVVQ QPVIGILRNT NDDFLAIGSG IYRSPSGDVD VNFHQVLIIG
Mias            NVFKKLVCRH PDNIHY-IKV DEFEYLTNVN DEELQAIVVQ QPVIGILRNT NRDFLGIGSG IYRSPSGDVD INFHQVLIIG
Zapa            NVFKKLVCRH PDKIHT-IKV DEFEYLTNVN DEELQAIVVQ QPVIGILRNT NDDFLAIGSG IYRSPSGDVD VNFHQVLIIG

Exon number     7777777777 7777777777 7777777777 7777777777 7777777777 77777777
                ....|....| ....|....| ....|....| ....|....| ....|....| ....|...
                     165        175        185        195        205        215
A. lyrata t2    YGYD--NGKP YWIIQNSYGE GWGNGGFGYV YRRIKSGQGS EFFAVAYPKI RGFPRKPR*
Klet            YGYDDSNGKP YWIIQNSYGE GWGNGGFGYV YRRINSGQGS EFFAVAYPKI RGYPRKPR*
Kowa            YGYD--NGKP YWIIQNSYGE GWGNGGFGYV YRRIKSGQGS VFFAVAYPKI RGFPRKQR*
Mias            YGYDDSNGKP YWIIQNSYGE GWGNGGFGYV YRRINSGQGS EFFAVAYPKI RGYPRKPR*
Zapa            YGYD--NGKP YWIIQNSYGE GWGNGGFGYV YRRIKSGQGS EFFAVAYPKI RGFPRKPR*
```

Figure S6. Alignment of the predicted variants of the *A. halleri* Cysteine Protease-Like 1 (CPL1) protein. Shown are the conceptually translated consensus coding sequences of AL8G32870 from two metallicolous (Klet and Mias) and two non-metallicolous (Kowa and Zapa) populations of *A. halleri* based on the *A. lyrata* genome annotation version 2.1 (transcript variant t2). Amino acids that are polymorphic among the five genotypes are printed in bold type, with those common to both metalliferous (M) sites shown in red fonts and those common to both non-metalliferous (NM) sites shown in blue fonts, and highlighted in yellow when M populations share derived alleles. A grey bar above the alignment marks the PF00112 papain family cysteine protease domain. Catalytic residues are underlined.
