# Supplementary material for: Convergent evolution in Arabidopsis halleri and Arabidopsis arenosa on calamine metalliferous soils
Source: Philos Trans R Soc Lond B Biol Sci. 2019 Jun 3;374(1777):20180243. doi: 10.1098/rstb.2018.0243 (PMC6560266; doi:10.1098/rstb.2018.0243)
Supplement: Figure S9 [file rstb20180243supp9.pdf]

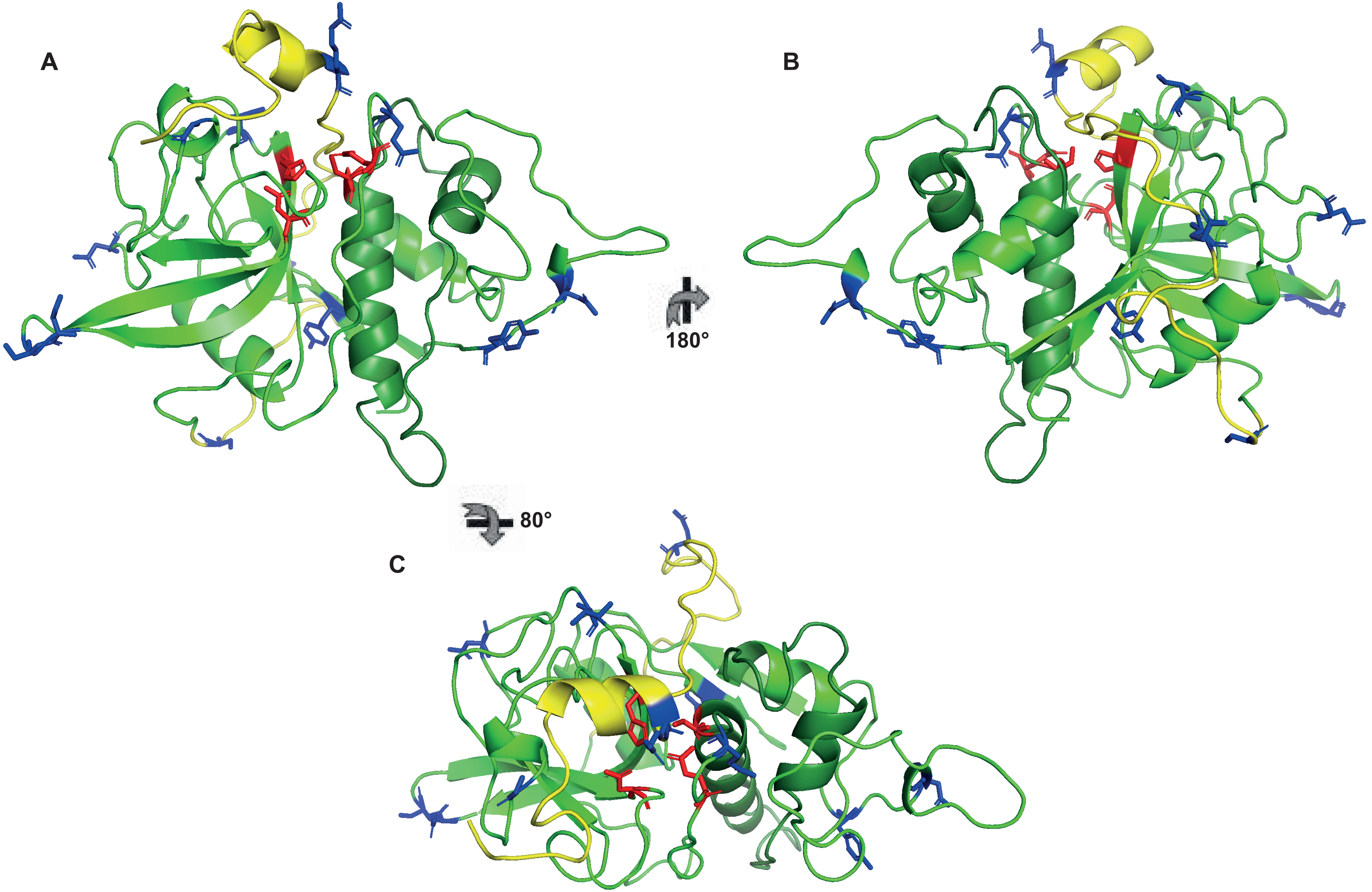

Figure S9. Predicted structure of the Mias AhCPL1 protein variant shown as a light green ribbon. The four catalytic residues are shown as red sticks. Residues which are derived in both metallicolous populations Mias and Klet are shown as blue sticks. The segment of the protein incorporated by additional *A. halleri* exonic sequence is shown in dark forest green. The Propeptide that is likely cleaved during enzyme maturation is shown in yellow. **A**, Front view. **B**, Back view. **C**, View looking down upon the catalytic crevice.
