# Supplementary material for: Convergent evolution in Arabidopsis halleri and Arabidopsis arenosa on calamine metalliferous soils
Source: Philos Trans R Soc Lond B Biol Sci. 2019 Jun 3;374(1777):20180243. doi: 10.1098/rstb.2018.0243 (PMC6560266; doi:10.1098/rstb.2018.0243)
Supplement: Supplementary methods [file rstb20180243supp10.docx]

**Supplementary methods**

(a) Plant cultivation under growth chamber conditions

Field-collected *A. arenosa* seeds were surface-sterilized in 1.5-mL polypropylene tubes. We incubated seeds in 1.5 mL 70% (v/v) ethanol (anal. grade) for 5 min, washed in 1 mL sterile ultrapure water for 1 min, followed by two 10 min incubations in 3.25% (w/v) NaOCl with 0.05% (v/v) Tween 20, and four washes with 1 mL sterile ultrapure water. The sterilized seeds were then transferred onto round polystyrene petri plates (9.2 cm Ø x 16 mm, Sarstedt, Nümbrecht, Germany), each filled with 40 mL 0.5x MS medium containing 1% (w/v) sucrose, and solidified with 1% (w/v) agar (type M), incubated at 4°C in the dark for 3 days, then placed vertically into a climate-controlled growth chamber for 10 days (22°C/18°C, 11 h light, 120 µmol m^-2^ s^-1^; Percival, CLF Plant Climatics, Emersacker, Germany). For vegetative propagation of *A. halleri* plants vegetative stolons (~ 2 cm in length) were cut from mother plants and transferred into peat:sand mix for rooting and pre-cultivation for 17 days (see Methods).

(b) DNA extraction from freeze-dried plant leaves

CTAB buffer was composed of 2% (w/v) CTAB, 10% (v/v) 1 M Tris pH 8.0, 28% (v/v) 5 M NaCl, 4% (v/v) 0.5 M EDTA pH 8.0, 0.1% (v/v) 2-mercaptoethanol [1] and it additionally contained 1.5% (w/v) PVP-40 and 1% (w/v) Na_2_S_2_O_5_. The samples were mixed with the CTAB buffer by vigorous vortexing for 1 min and then incubated for 60 min at 65°C. We added 0.9 mL chloroform (containing 4% v/v isoamylalcohol), mixed gently and centrifuged at 8000 *g* for 10 min at room temperature. The aqueous layer was recovered (~0.75 mL) and mixed with 5 µL of 10 mg mL^-1^ RNAse A. and incubated for 20 min at 37°C followed by an extraction with 0.75 mL phenol:chloroform mix (50% v/v phenol (TE-buffered pH 7.5-8.0), 48% v/v chloroform, 2% v/v isoamylalcohol) and centrifugation at 8000 *g* for 10 min at 10°C. The aqueous phase (0.6 mL) was mixed by inverting 45 times with 0.48 mL isopropanol and centrifuge at 8000 g for 15 min at 4°C for precipitation of DNA. The pellet was washed twice with 0.4 mL ice-cold 80% (v/v) ethanol (anal. grade) and air-dried at RT for 5 min before addition of 40 µL T0.1E (10 mM Tris-HCl, 0.1 mM EDTA, pH 7.6) and gentle mixing.

(c) Statistical analysis of environmental and phenotypic data

To test whether *A. halleri* and *A. arenosa* share the same edaphic microhabitats at a common site, we performed one-way ANOVA on linear models of the form “concentration of element / proton ~ species” in R [2]. We tested for normal distribution of the residuals using the Shapiro test and for homoscedasticity of the variants using the Breusch-Pagan test (R-package olsrr [3]). In case of violation of these assumptions, we log or rank (base functions in R; rank with ties.method set to random) transformed the corresponding variable prior to ANOVA (R-package MASS [4]). To select elements that differ significantly between at least one metalliferous and both non-metalliferous populations, we performed a one-way ANOVA on linear models of the form “concentration of element / proton ~ populations” in R [2]. The same procedure was performed to test normal distribution and homoscedasticity. In case of violation of these assumptions, we Box-Cox transformed [5] the corresponding variable prior to ANOVA (R-package MASS [4])

(d) Library preparation and next generation sequencing (NGS)

For library preparation, we used 300 to 500 ng of gDNA (instead of 1 µg). Libraries were quantified using a Qubit dsDNA HS assay with an incubation time of 20 min. We obtained 125-bp paired-end reads on a Illumina’s HiSeq 2500 platform. Multiplexing of libraries was done as advised by Illumina; however, instead of quantifying the PCR-free libraries using qPCR, we ran a “quant lane”, that is, we multiplexed up to 96 samples and sequenced one lane. According to the yield of the quant lane, our consecutive sequencing strategy involved iterative sequencing runs until we reached a minimum of 10-fold average coverage for each sample, based on the number of raw reads. In total, we obtained 192x10^9^ bp and 327x10^9^ bp raw sequence data for *A. halleri* and *A. arenosa*, respectively.

(e) Initial processing of NGS data, alignment and variant discovery

**Genome scan and large-effect variant identification pipeline**

Adapter sequences were clipped from the ends of the reads using the program cutadapt 1.14 [6], allowing ≤ 0.15 sequence divergence (error rate) over ≥ 4 bp in length for identifying adapters. Processed reads of ≥ 25 bp were maintained and aligned to the *Arabidopsis lyrata* reference genome [7] using the program bwa mem version 0.7.15 [8], with a seed size of *k* = 10 for an initial match of 10 bp length. The resulting SAM file was converted into a BAM (htslib of Samtools 1.3.1) [9]. Duplicate reads were removed with 2.7.1 MarkDuplicates function (picard-tools, http://broadinstitute.github.io/picard). The AddOrReplaceReadGroups function (picard-tools) was used to assign reads to read groups corresponding to samples. The RealignerTargetCreator and the IndelRealigner tools of GATK 3.7 [10-12] were then used to realign regions that had been poorly aligned in the initial alignment.

The GATK HaplotypeCaller was used to call single nucleotide polymorphisms (SNPs) and insertions/deletions (indels) in relation to the reference genome for each sample, with settings of a minimum base quality score of 25, a minimum mapping quality score of 25, an expected heterozygosity of 0.02 for *A. halleri* and 0.04 for *A. arenosa*, expected indel heterozygosity of 0.01 for *A. halleri* and 0.02 for *A. arenosa*, and ploidy of 2 for *A. halleri* and 4 of *A. arenosa*. We employed the read filters DuplicateRead, BadMate and BadCigar with Emitting Reference Confidence scores (ERC) set to BP_Resolution. For all samples of one species from a given population pair, joint genotyping and calculation of allele frequencies was performed using the GATK GenotypeGVCFs choosing newQual, as well as SNP and indel heterozygosities as in the HaplotypeCaller.

In order to be able to cross-validate potentially high-effect variants, all SNPs were additionally called within each population pair by the UnifiedGenotyper from GATK 3.7, with a minimum base quality score of 25, a standard minimum confidence threshold for calling of 25, a downsampling to a coverage of 200, heterozygosity and ploidy settings and read filters as specified above, with the genotype likelihood model set to SNP. For indels, we additionally used the program GROM [13] on the realigned data retaining variants with settings as used in the GATK HaplotypeCaller, retaining only those indels with a minimum standard deviation for discordance of 30. Indels were retrieved from the output with “grep "SPR:SEV:SRD:SCO:ECO:SOT
:EOT:SSC:HP\|SPR:EPR:SEV:EEV:SRD:ERD:SCO:ECO:SOT:EOT:SSC:ESC:HP"”.

SNPs and Indels were filtered separately using the GATK VariantFiltration, SelectVariants and VariantsToTable. SNP and indel filtering parameters were set based on GATK best practices recommendations, “hard filtering” [11]. We retained SNPs and indels that were biallelic with no missing genotypes. For SNPs, we additionally required a minimum QualByDepth (QD) of 2, maximum FisherStrand (FS) of 40, minimum RMSMappingQuality (MQ) of 50, minimum MappingQualityRankSumTest (MQRankSum) of -2.5, minimum ReadPosRankSumTest (ReadPosRankSum) of -4 and maximum StrandOddsRatio (SOR) of 4. For indels, we additionally required a minimum QD of 20, maximum FS of 40, minimum ReadPosRankSum of -4 and maximum SOR of 4. For both SNPs and indels, the minimum depth of coverage was set to 2, and subsequently all sites with a coverage ≤ the 2.5 percentile and ≥ 97.5 percentile were excluded. Variants were then assigned to their respective populations and vcf files transformed into tables. The site frequency spectra were visualized with the hist function in R showing the absolute allele frequencies of all SNPs.

**Bayenv2/EAA Pipeline**

To prepare the raw sequencing data for mapping we concatenated the fastq.gz files from the different sequencing lanes, followed by removing adapter sequence from reads that had inserts shorter than 250 bp, using cutadapt 1.9 [6]. We mapped both species to a North American *Arabidopsis lyrata* reference genome [7] using bwa mem [9]. We concluded this to be a valid approach as 88.3 % and 90.0 % of reads mapped to this reference for *A. arenosa* and *A. halleri*, respectively. We assume a similar phylogenetic distance of *A. arenosa* and *A. halleri* from *A. lyrata,* thereby avoiding a mapping bias. Next, we marked duplicate reads using “MarkDuplicates” from picard-tools 1.134 [11]. Using picard-tools’ “AddOrReplaceReadGroups” we added read groups and indices to the sequencing bam files. The last step of the mapping part of the processing pipeline was to re-align the reads to accommodate indels using the GATK tools “RealignerTargetCreator” and “IndelRealigner” [10]. These steps created analysis-ready bam files. Before we proceeded to discover variants in the analysis-ready bam files, we assessed the depth of coverage in our sample set by using the GATK tool “DepthOfCoverage” (DOC) with the restriction to a minimum base quality of 25 and a minimum mapping quality of 25 (both values are on a PHRED scale). We restricted the calculation of DOC to the annotated gene space and excluded all individuals with < 70% of bases with a minimum DOC of 4.

After this DOC selection we had 31 *A. arenosa* and 34 *A. halleri* individuals from two metalliferous (Klet, Mias) and two non-metalliferous (Kowa, Zapa/Zako) populations. We used the GATK tool “HaplotypeCaller” to discover and call genetic variants (SNPs, indels and multi-nucleotide-polymorphisms) for each individual. To speed up the process, we ran the eight main scaffolds of the *A. lyrata* reference genome in parallel. We specified output at bp-resolution: that is each single base is listed in the GVCF. For better organisation, we combined all individuals of one species of one population into multisample GVCFs using “CombineGVCFs”. From this stage onwards, the two sibling-species were processed separately from each other. The last step of the variant discovery was joint genotyping by using the GATK tool “GenotypeGVCFs”. Because all samples are genotyped together, rare variants are more readily identified, rather than mistaken for sequencing errors.

For the DivergenceScan and environmental association analysis (EAA), we filtered the VCFs in the following way: First, we excluded all indel and mixed sites, restricting the remaining sites to be biallelic, using the GATK tool “SelectVariants”. Second, we flagged sites that were either of QD < 2.0, FS > 60.0, MQ < 40.0, MQRankSum <- 12.5, ReadPosRankSum < -8.0 or HaplotypeScore < 13.0, using the tool “VariantFiltration”. We filtered for each quality criterion by a separate command to ensure that we flag any site that failed at least one of the criteria. Third, to exclude potential paralogous sites and avoid potential conflicts of different genetic variants between paralogs, we masked sites that had excess read depth that we defined as 1.6x the mode of the read depth distribution across all individuals for one species. For the lower read depth cutoff we took the read depth that covers each sequenced chromosome at least once (DP < 124, DP > 692 for autotetraploid *A. arenosa* and DP < 68, DP > 719 for diploid *A. halleri*). Excess read depth was flagged using the GATK tool “VariantFiltration”. Fourth, we excluded all sites that failed any of the above filters, using “SelectVariants”. Fifth, we split the data into cohorts depending on desired contrasts. We used “SelectVariants” to extract the cohorts from the filtered VCF. Sixth, we export the allele counts per cohort using the tool “VariantsToTable”. The allele count tables per cohort are the basis for the following analyses of DivergenceScan and EAA (see section (i)).

(f) Genome Scans, large-effect variant identification, candidate gene lists and convergent evolution

Analyses were performed in R version 3.4.4. The genome scans were done using custom R scripts (https://github.com/syllwlwz/Divergence-Scans). For each population pair, the genome was partitioned into windows of 25 consecutive SNPs, for which we calculated the pairwise metrics Differentiation-Diversity residuals (DD) [14, 15] Wright’s Fixation Index (F_ST_) [14, 16, 17], Two-Dimensional Site Frequency Spectrum Composite Likelihood Ratio test (Nielsen 2dSFS) [14, 15, 18], Maximum Absolute Net Divergence (d_XY_) [14, 19], and T_F-LK_’ *P*-value (extended Lewontin and Krakauer test, Flk) [20], VarLD [21]. Flk was calculated using the software hapflk version 1.3.0 [22]. For *A. arenosa*, the heterozygosity was adjusted to $4p^{3q}+6p^{2q^{2}}+4\mathrm{pq}^{3}$ for F_ST_ and Flk. The 25-SNP windows were ranked for each metric using the rank function, with the “ties” option set to minimum. The single population metrics Tajima’s D [23] and Fay and Wu’s H [24] were calculated for windows of 25 consecutive SNPs based on within-population sequence variation. Tajima’s D and Fay and Wu’s H were standardized genome- and population-wide employing the function decostand using the method “standardize” in the R-package vegan [25]. File format was converted from vcf to bed with GATK VariantsToBinaryPed, and SweeD [26, 27] was calculated with the program SweeD with a grid size of 100,000 for *A. halleri* and 200,000 for *A. arenosa* and a folded site frequency spectrum. Windows exceeding 100,000 bp in length were excluded for both pairwise and the single population metrics.

Candidate windows for selection were identified as the 0.1%ile for either one of the pairwise metrics F_ST_, DD, Nielsen 2dSFS, Flk or d_XY_ (see also later filtering). Tajima’s D, Fay and Wu’s H and SweeD nearest windows were associated with candidate windows using the “nearest” command in the R-package GenomicRanges [28] For the population pair Klet and Kowa of *A. halleri*, DD results were not taken into consideration because the pre-requisite of a positive correlation of diversity with divergence was not fulfilled. VarLD outliers were not considered because the underlying assumption of high linkage disequilibrium (LD) at selected loci appeared not to be fulfilled.

Employing positional information according to the *A. lyrata* reference genome, each window was converted to its gene content based on any overlap with the coding region and up to 2,000 bp upstream (promoter region), with the R-packages GenomicRanges, GenomicFeatures and IRanges [28]. Orthologous *Arabidopsis thaliana* gene identifiers were retrieved for *A. lyrata* genes based on an orthogroup analysis through OrthoFinder [https://github.com/davidemms/OrthoFinder] [29]. Each OrthoGroup contains genes that are inferred to share a recent common ancestor prior to speciation. Genes not assigned to an OrthoGroup comprise species-specific genes as well as incorrect annotations. Primary transcripts of protein coding genes for *A .thaliana* (TAIR10) (www.arabidopsis.org; May 24, 2017) and *A. lyrata* [30] were downloaded from Phytozome (https://phytozome.jgi.doe.gov/pz/portal.html; May, 2017; [31]).

Genes lacking an *A. thaliana* orthologue were submitted for a local blastx on the nr database [32], choosing the "7 qseqid sseqid pident length mismatch gapopen qstart qend sstart send evalue bitscore stitle" option for the output format with a maximum number of high scoring pairs (HSPs) of 1, a maximum number of aligned sequences of 1 and all “Arabidopsis“-containing entries in the species field excluded from the database with the negative_gilist option.

All variants (SNPs and indels) were annotated and their effects predicted by SnpEff [33] based on the *Arabidopsis lyrata* annotation version 2 [30]. Genome scan metrics and allele frequency plots over candidate genomic regions were generated with custom R scripts for manual inspection of the selected candidates for each population pair. Metrics plots were rated from A (most promising) to C (likely false positive) based on the following criteria in the assessment of 25-SNP windows (for A): (i) The 0.1%ile of the respective pairwise metric(s) was reached within the candidate gene (or its promoter region); (ii) the 0.1%ile of the pairwise metric(s) did not extend over a larger region comprising two or more entire consecutive genes; (iii) at least one pairwise set of single-population metrics was consistent with positive selection in the metalliferous population. Allele frequency plots were assigned manually to classes A (most promising) to D (false positive), based on the following criteria (for A): (i) Several local SNPs exhibited an absolute allele frequency difference > ~0.7; (ii) pronounced allele frequency differences did not extend over a larger region comprising two or more entire genes; (iii) high abundance of non-synonymous SNPs in the candidate gene. Genes in the bottom class for either one of these two assessments were eliminated from the group of candidate genes. In a further step, we maintained only those candidate genes for which (a) the standardized Tajima’s D or Fay and Wu’s H value of the closest 25 SNP window was negative, or the SweeD Likelihood was positive in the metalliferous population, and (b) values were more extremely negative or positive, respectively, in the metalliferous population for at least one of these three metrics. Metrics and allele frequency plot ratings and single population metrics values are documented in supplemental datasets S2, S3, S4, and S5.

In addition to the evaluation of candidate genes from genome scans, the genome-wide output of SnpEff (see above) was used to identify large-effect variants. We chose SNPs called by both HaplotypeCaller and UnifiedGenotyper and predicted by SnpEff to have a “high impact”, i.e., generate a frameshift or a stop codon. Furthermore, we chose indels called by both the HaplotypeCaller and GROM, with the predicted indel positions differing by at most the length of the indel and predicted by SnpEff to have a “high impact”. Based on the identified variants, we considered as candidates genes for containing selected large-effect variants those genes for which SNPs and indels exhibited a minimum allele frequency difference of 0.4 and an allele frequency of ≥ 0.9 or ≤ 0.1 in the population at the metalliferous site. Genes with different high effect mutations and allele frequencies of one higher in the one population of a pair and of another higher in another population were excluded.

To verify that the identified candidates genes were neither duplicated nor sequenced at insufficient depth, coverage was calculated from the realigned bam files with bedtools’ coverage function [34] based on the *A. lyrata* reference genome, with a histogram output. The histogram output was then converted into a calculable input with custom R scripts using the data.table package [35], and the mean and median calculated per gene or per exonic gene content. In addition, to earmark genes with potential paralogues in the genome, the number of paralogous genes within the same orthogroup was extracted from the OrthoFinder results for *A. halleri* [36], *A. lyrata* and *A.* *thaliana*, respectively, with the data.table package within custom R scripts.

To identify common candidate genes, the function “merge” was used with the parameter “by” set to the *Arabidopsis lyrata* gene identifier to merge datasets for all combinations of population pairs. To visualize these, Venn diagrams of candidate genes were generated with Venny 2.1.0 [37] based on the *Arabidopsis lyrata* gene identifiers. To compare observed and expected gene numbers, hypergeometric tests were performed using the phyper function with lower.tail = F and log.p = FALSE to calculate the probability of finding this many or more genes in the overlap. Inputs were (i) the number of genes in common between two population pairs reduced by one, (ii) the number of genes in the first population pair, (iii) the number of genes in the second population pair, and (iv) the difference between the total number of genes annotated in the *A. lyrata* genome (33,221) and the number of genes in the first population pair (https://github.com/syllwlwz/Divergence-Scans/blob/master/Genome_scan
_analysis/hypergeom_convergent_evol.R, supplemental table S5). To account for possible species-specific genes or genes with missing data in one of the two compared population pairs, the test was repeated employing an approximated minimum total number of genes of 25,000, and all statistically significant observations were robust to this change (see Figure 3A).

A gene function enrichment test was performed for each population pair using the ClueGO app version 2.5.2 [38] in Cytoscape version 3.6.1 [39] using the *A. thaliana* gene identifiers and gene ontology (GO) BiologicalProcess 21.11.2017 including 22,365 available unique genes and 5,480 terms. GO Term Fusion was enabled to fuse GO parent-child terms based on similar associated genes and only pathways with a p-value below or equal to 0.05 were kept. The network specificity was kept at the default medium. The enrichment test was computed for all candidates without the additional candidate genes containing large-effect indels and SNPs.

(i) Identifying divergence signatures, EAA, and compilation of candidate gene lists

We restricted the length of the SNP-based windows for population genetic metrics calculations to be shorter than the longest gene in the *A. lyrata* annotation [30]. We did this to avoid poorly annotated regions and regions largely deprived of SNPs, such as the centromeric region.

To identify the most diverged windows, we wrote the python3 pipeline DivergenceScan (https://github.com/SailerChristian/Divergence_Scan). In the DivergenceScan, we first selected the windows that are ≥ 99.5%ile of the empirical distributions for each metric. Second, to identify the genes the identified divergence windows are part of, we used bedtools’ “intersect" [34, 40], specifying that the divergence window must overlap by at least 1 base with an annotated gene coding locus. Those genes are the DivergenceScan candidate genes.

The DivergenceScan candidate gene list is purely based on genomic data and therefore contains genetic differences that are due to causes other than only soil elemental driven selective pressure, such as genetic drift. Therefore, we tested the divergence scan candidates for association to environmental variables using Bayenv2 [41], which allows for testing in a two-step process. After calculating the median per population of the soil trace metal element (TME) concentrations we log transformed them and used the standardized values. The first step of Bayenv2 is to calculate the variance-covariance matrix that corrects for neutral population structure using presumably neutral four-fold degenerated sites. Second, we extracted the SNPs for all DivergenceScan candidate genes and converted the file format to Bayenv2 input format using a custom python3 script. The second step of Bayenv2 is to calculate the Bayes factor (BF), Spearman’s ρ and Pearson’s correlation coefficient r_s_, which is the actual EAA. We used 100 000 iterations.

To annotate the effects of the found SNPs with SnpEff [33], we added the *A. lyrata* v2.0 annotation to the SnpEff database and annotated the corresponding SNPs only (no upstream or downstream). Next, we combined per SNP allele frequency, d_XY_ and F_ST_ data with the SnpEff annotation and the results from Bayenv2. We selected only SNPs that have BF ≥ 100 [13] for the selected elements (environmentally associated SNP, EA SNP) and further required that the EA SNP causes a non-synonymous change. We did this per contrast divergence scan candidate gene list and species (four lists).

References

1. Rogstad, S. H. 1992 Saturated NaCl-CTAB Solution as a Means of Field Preservation of Leaves for DNA Analyses. *Taxon* **41**, 701-708. (DOI:10.2307/1222395).

2. R Development Core Team. 2008 *R: A language and environment for statistical computing.* Vienne, Austria, R Foundation for Statistical Computing.

3. Hebbali, A. 2018 olsrr: Tools for Building OLS Regression Models. R package version 0.5.0.

4. Venables, W. N. & Ripley, B. D. 2002 *Modern Applied Statistics with S*. Fourth Edition ed. New York, Springer.

5. Box, G. E. P. & Cox, D. R. 1964 An Analysis of Transformations. *Journal of the Royal Statistical Society. Series B (Methodological)* **26**, 211-252.

6. Martin, M. 2011 Cutadapt removes adapter sequences from high-throughput sequencing reads. *2011* **17**, 3. (DOI:10.14806/ej.17.1.200).

7. Hu, T. T., Pattyn, P., Bakker, E. G., Cao, J., Cheng, J. F., Clark, R. M., Fahlgren, N., Fawcett, J. A., Grimwood, J., Gundlach, H., et al. 2011 The *Arabidopsis lyrata* genome sequence and the basis of rapid genome size change. *Nat Genet* **43**, 476-481. (DOI:10.1038/ng.807).

8. Li, H. & Durbin, R. 2010 Fast and accurate long-read alignment with Burrows-Wheeler transform. *Bioinformatics* **26**, 589-595. (DOI:10.1093/bioinformatics/btp698).

9. Li, H. & Durbin, R. 2009 Fast and accurate short read alignment with Burrows-Wheeler transform. *Bioinformatics* **25**, 1754-1760. (DOI:10.1093/bioinformatics/btp324).

10. McKenna, A., Hanna, M., Banks, E., Sivachenko, A., Cibulskis, K., Kernytsky, A., Garimella, K., Altshuler, D., Gabriel, S., Daly, M., et al. 2010 The Genome Analysis Toolkit: a MapReduce framework for analyzing next-generation DNA sequencing data. *Genome Res* **20**, 1297-1303. (DOI:10.1101/gr.107524.110).

11. DePristo, M. A., Banks, E., Poplin, R., Garimella, K. V., Maguire, J. R., Hartl, C., Philippakis, A. A., del Angel, G., Rivas, M. A., Hanna, M., et al. 2011 A framework for variation discovery and genotyping using next-generation DNA sequencing data. *Nat Genet* **43**, 491-498. (DOI:10.1038/ng.806).

12. Van der Auwera, G. A., Carneiro, M. O., Hartl, C., Poplin, R., Del Angel, G., Levy-Moonshine, A., Jordan, T., Shakir, K., Roazen, D., Thibault, J., et al. 2013 From FastQ data to high confidence variant calls: the Genome Analysis Toolkit best practices pipeline. *Curr Protoc Bioinformatics* **43**, 11 10 11-33. (DOI:10.1002/0471250953.bi1110s43).

13. Smith, S. D., Kawash, J. K. & Grigoriev, A. 2017 Lightning-fast genome variant detection with GROM. *Gigascience* **6**, 1-7. (DOI:10.1093/gigascience/gix091).

14. Arnold, B. J., Lahner, B., DaCosta, J. M., Weisman, C. M., Hollister, J. D., Salt, D. E., Bomblies, K. & Yant, L. 2016 Borrowed alleles and convergence in serpentine adaptation. *PNAS* **113**, 8320-8325. (DOI:www.pnas.org/cgi/doi/10.1073/pnas.1600405113).

15. Yant, L., Hollister, J. D., Wright, K. M., Arnold, B. J., Higgins, J. D., Franklin, F. C. H. & Bomblies, K. 2013 Meiotic adaptation to genome duplication in *Arabidopsis arenosa*. *Curr Biol* **23**, 2151-2156. (DOI:10.1016/j.cub.2013.08.059).

16. Ross-Ibarra, J., Wright, S. I., Foxe, J. P., Kawabe, A., DeRose-Wilson, L., Gos, G., Charlesworth, D. & Gaut, B. S. 2008 Patterns of polymorphism and demographic history in natural populations of *Arabidopsis lyrata*. *PLoS One* **3**, e2411. (DOI:10.1371/journal.pone.0002411).

17. Wright, S. 1951 The genetical structure of populations. *Ann Eugen* **15**, 323-354.

18. Nielsen, R., Hubisz, M. J., Hellmann, I., Torgerson, D., Andres, A. M., Albrechtsen, A., Gutenkunst, R., Adams, M. D., Cargill, M., Boyko, A., et al. 2009 Darwinian and demographic forces affecting human protein coding genes. *Genome Res* **19**, 838-849. (DOI:10.1101/gr.088336.108).

19. Smith, J. & Kronforst, M. R. 2013 Do Heliconius butterfly species exchange mimicry alleles? *Biol Lett* **9**, 20130503. (DOI:10.1098/rsbl.2013.0503).

20. Bonhomme, M., Chevalet, C., Servin, B., Boitard, S., Abdallah, J., Blott, S. & SanCristobal, M. 2010 Detecting Selection in Population Trees: The Lewontin and Krakauer Test Extended. *Genetics* **186**, 241-262. (DOI:10.1534/genetics.110.117275).

21. Teo, Y. Y., Fry, A. E., Bhattacharya, K., Small, K. S., Kwiatkowski, D. P. & Clark, T. G. 2009 Genome-wide comparisons of variation in linkage disequilibrium. *Genome Res* **19**, 1849-1860. (DOI:10.1101/gr.092189.109).

22. Fariello, M. I., Boitard, S., Naya, H., SanCristobal, M. & Servin, B. 2013 Detecting signatures of selection through haplotype differentiation among hierarchically structured populations. *Genetics* **193**, 929-941. (DOI:10.1534/genetics.112.147231).

23. Tajima, F. 1989 Statistical Method for Testing the Neutral Mutation Hypothesis by DNA Polymorphism. *Genetics* **123**, 585-595.

24. Fay, J. C. & Wu, C.-I. 2000 Hitchhiking Under Positive Darwinian Selection. *Genetics* **155**, 1405-1413.

25. Oksanen, J., Blanchet, F. G., Friendly, M., Kindt, R., Legendre, P., McGlinn, D., Minchin, P. R., O'Hara, R. B., Simpson, G. L., Solymos, P., et al. 2018 vegan: Community Ecology Package.

26. Nielsen, R., Williamson, S., Kim, Y., Hubisz, M. J., Clark, A. G. & Bustamante, C. 2005 Genomic scans for selective sweeps using SNP data. *Genome Research* **15**, 1566-1575. (DOI:10.1101/gr.4252305).

27. Pavlidis, P., Zivkovic, D., Stamatakis, A. & Alachiotis, N. 2013 SweeD: likelihood-based detection of selective sweeps in thousands of genomes. *Mol Biol Evol* **30**, 2224-2234. (DOI:10.1093/molbev/mst112).

28. Lawrence, M., Huber, W., Pages, H., Aboyoun, P., Carlson, M., Gentleman, R., Morgan, M. T. & Carey, V. J. 2013 Software for computing and annotating genomic ranges. *PLoS Comput Biol* **9**, e1003118. (DOI:10.1371/journal.pcbi.1003118).

29. Emms, D. M. & Kelly, S. 2015 OrthoFinder: solving fundamental biases in whole genome comparisons dramatically improves orthogroup inference accuracy. *Genome Biol* **16**, 157. (DOI:10.1186/s13059-015-0721-2).

30. Rawat, V., Abdelsamad, A., Pietzenuk, B., Seymour, D. K., Koenig, D., Weigel, D., Pecinka, A. & Schneeberger, K. 2015 Improving the Annotation of *Arabidopsis lyrata* Using RNA-Seq Data. *PLoS One* **10**, e0137391. (DOI:10.1371/journal.pone.0137391).

31. Goodstein, D. M., Shu, S., Howson, R., Neupane, R., Hayes, R. D., Fazo, J., Mitros, T., Dirks, W., Hellsten, U., Putnam, N., et al. 2012 Phytozome: a comparative platform for green plant genomics. *Nucleic Acids Res* **40**, D1178-1186. (DOI:10.1093/nar/gkr944).

32. Altschul, S. F. 1990 Basic Local Alignment Search Tool. *J. Mol. Biol.* **215**, 403-410. (DOI:0022-2836/90/190403-08).

33. Cingolani, P., Platts, A., Wang le, L., Coon, M., Nguyen, T., Wang, L., Land, S. J., Lu, X. & Ruden, D. M. 2012 A program for annotating and predicting the effects of single nucleotide polymorphisms, SnpEff: SNPs in the genome of *Drosophila melanogaster* strain w1118; iso-2; iso-3. *Fly (Austin)* **6**, 80-92. (DOI:10.4161/fly.19695).

34. Quinlan, A. R. & Hall, I. M. 2010 BEDTools: a flexible suite of utilities for comparing genomic features. *Bioinformatics* **26**, 841-842. (DOI:10.1093/bioinformatics/btq033).

35. Dowle, M. & Srinivasan, A. 2017 data.table: Extension of `data.frame`.

36. Briskine, R. V., Paape, T., Shimizu-Inatsugi, R., Nishiyama, T., Akama, S., Sese, J. & Shimizu, K. K. 2017 Genome assembly and annotation of *Arabidopsis halleri*, a model for heavy metal hyperaccumulation and evolutionary ecology. *Mol Ecol Resour* **17**, 1025-1036. (DOI:10.1111/1755-0998.12604).

37. Oliveros, J. C. 2007-2015 Venny. An interactive tool for comparing lists with Venn´s diagrams. http://bioinfogp.cnb.csic.es/tools/venny/index.html.

38. Bindea, G., Mlecnik, B., Hackl, H., Charoentong, P., Tosolini, M., Kirilovsky, A., Fridman, W. H., Pages, F., Trajanoski, Z. & Galon, J. 2009 ClueGO: a Cytoscape plug-in to decipher functionally grouped gene ontology and pathway annotation networks. *Bioinformatics* **25**, 1091-1093. (DOI:10.1093/bioinformatics/btp101).

39. Shannon, P., Markiel, A., Ozier, O., Baliga, N. S., Wang, J. T., Ramage, D., Amin, N., Schwikowski, B. & Ideker, T. 2003 Cytoscape: A Software Environment for Integrated Models of Biomolecular Interaction Networks. *Genome Research* **13**, 2498-2504.

40. Quinlan, A. R. 2014 BEDTools: The Swiss-Army Tool for Genome Feature Analysis. *Curr Protoc Bioinformatics* **47**, 11 12 11-34. (DOI:10.1002/0471250953.bi1112s47).

41. Gunther, T. & Coop, G. 2013 Robust identification of local adaptation from allele frequencies. *Genetics* **195**, 205-220. (DOI:10.1534/genetics.113.152462).
