# Supplementary material for: Convergent evolution in Arabidopsis halleri and Arabidopsis arenosa on calamine metalliferous soils
Source: Philos Trans R Soc Lond B Biol Sci. 2019 Jun 3;374(1777):20180243. doi: 10.1098/rstb.2018.0243 (PMC6560266; doi:10.1098/rstb.2018.0243)
Supplement: Table S1 [file rstb20180243supp11.docx]

| **Table S1.** Sampling, sequencing and sequence data analysis details for *Arabidopsis halleri* and *Arabidopsis arenosa* | | | | | | | | | | | |  |
| --- | --- | --- | --- | --- | --- | --- | --- | --- | --- | --- | --- | --- |
| **Population** | **Longitude** | **Latitude** | **Altitude**[m.a.s.l.] | **Soil Type** | **No. of individuals  *A. halleri /***  ***A. arenosa*** | **Mapping efficiency**^§^ **[%] *A. halleri* /  *A.* *arenosa*** | **Depth of genome coverage**^$^ **[fold] *A. halleri* / *A. arenosa*** | **No. filtered SNPs**^1^  ***A. halleri* / *A. arenosa*** | **No. 25-SNP-windows**^1^  ***A. halleri* / *A. arenosa*** | **No. SNPs tested in EAA**^2^  ***A. halleri* / *A. arenosa*** | **% SNPs**  **BF ≥ 100**^2^  ***A. halleri* / *A. arenosa*** | |
| Miasteczko Śląskie (Mias) | E 18.94172 | N 50.50211 | 294 | M | 8 / 7^3^ | 84 / 85^1^ | 18 / 13^1,2^ | 2,028,889 / 4,710,179 | 81,137 / 188,371 |  |  | |
|  |  |  |  |  | 8 / 7 | 73 / 71^2^ | 16 / 13^2^ | 2,506,361 / 6,510,265^2^ |  | 112,608 / 537,422 | 0.78 / 0.192 | |
| Zakopane (Zapa) | E 19.96661 E 19.87836 | N 9.27892^a^  N 9.27408^h^ | 955 1006 | NM | 9 / 8^3^ | 81 / 86^1^ | 9 / 10^1,2^ |  |  |  |  | |
|  |  |  |  |  | 9 / 8 | 72 / 73^2^ | 9 / 10^2^ |  |  |  |  | |
| Kletno (Klet) | E 16.84842 | N 50.24408 | 746 | M | 10 / 9^3^ | 82 / 88^1^ | 11 / 14^1,2^ | 1,828,190 / 4,799,745 | 73,109 / 191,957 |  |  | |
|  |  |  |  |  | 10 / 8 | 72 / 76^2^ | 11 / 13^2^ | 2,308,543 / 5,747,551^2^ |  | 91,074 / 544,317 | 1.12 / 0.047 | |
| Kowary (Kowa) | E 15.84389 | N 50.76317 | 651 | NM | 7 / 8^3^ | 83 /88^1^ | 15 / 14^1,2^ |  |  |  |  | |
|  |  |  |  |  | 7 / 8 | 72 / 74^2^ | 15 / 14^2^ |  |  |  |  | |
| ^§^Mapping efficiency is based on proper read pairs (both reads mapped at expected distance in the expected orientation) ^$^Depth of coverage was calculated across coding regions ^a^Population of *A. arenosa* and *A. halleri* at Zakopane (Zapa; Stein *et al*., 2017) ^h^All *A. halleri* individuals sampled newly in this study were from the Zakopane site named Zako (as defined by Stein *et al*., 2017), because there was an insufficient number of *A. halleri* individuals at the Zakopane site Zapa (as defined by Stein et al., 2017). For simplicity, the name Zapa is used in this study to refer to both of these Zakopane sites.  ^1^genome scans with manual filtering, large-effect indels and SNPs;  ^2^EAA  ^3^Sequenced individuals specified in Supplementary dataset S1. In addition, we sequenced the following *A. halleri* individuals: Zako_002_h05, Mias_002_h19 and individuals that were collected earlier (Stein *et al*., 2017): Kowa_02, Kowa_04, Mias_03, Mias_05, Zako_h01 | | | | | | | | | | | | |

| **Table S2.** Statistical analysis of soil data  (*a*) ANOVA of the linear model Response variable ~ species for the Mias site | | | | | | | |
| --- | --- | --- | --- | --- | --- | --- | --- |
| **Element tested** | **transformation of  responsive value** |  | ***df*** | **SS** | **MS** | ***F*** | ***P*** |
| Exch. Al | no | species | 1 | 21.7 | 21.7 | 0.507 | 0.494 |
|  |  | residuals | 9 | 385.3 | 42.8 |  |  |
| Exch. B | no | species | 1 | 0.000028 | 0.000028 | 0.039 | 0.847 |
|  |  | residuals | 9 | 0.00647 | 0.000719 |  |  |
| Exch. Ca | no | species | 1 | 8.38 | 8.38 | 0.000807 | 0.978 |
|  |  | residuals | 9 | 93388 | 10377 |  |  |
| Exch. Cd | no | species | 1 | 0.0914 | 0.0914 | 0.012 | 0.9147 |
|  |  | residuals | 9 | 67.8279 | 7.536 |  |  |
| Exch. Cr | no | species | 1 | 0.0000063 | 0.0000063 | 0.476 | 0.508 |
|  |  | residuals | 9 | 0.0001188 | 0.0000132 |  |  |
| Exch. Cu | log | species | 1 | 1.52 | 1.52 | 0.850 | 0.381 |
|  |  | residuals | 9 | 16.1 | 1.79 |  |  |
| Exch. Fe | no | species | 1 | 30.8 | 30.8 | 0.819 | 0.389 |
|  |  | residuals | 9 | 338.6 | 37.6 |  |  |
| Exch. K | no | species | 1 | 34.17 | 34.17 | 0.453 | 0.518 |
|  |  | residuals | 9 | 678.8 | 75.42 |  |  |
| Exch. Mg | no | species | 1 | 0.339 | 0.339 | 0.00449 | 0.948 |
|  |  | residuals | 9 | 679.1 | 75.5 |  |  |
| Exch. Mn | log | species | 1 | 0.0346 | 0.0346 | 0.062 | 0.809 |
|  |  | residuals | 9 | 5.0289 | 0.5588 |  |  |
| Exch. Ni | no | species | 1 | 0.0000236 | 0.0000236 | 0.0393 | 0.847 |
|  |  | residuals | 9 | 0.00539 | 0.000599 |  |  |
| Exch. P | no^a^ | species | 1 | 3.57 | 3.57 | 3.436 | 0.101 |
|  |  | residuals | 8 | 8.313 | 1.039 |  |  |
| Exch. Pb | log | species | 1 | 0.856 | 0.856 | 0.673 | 0.433 |
|  |  | residuals | 9 | 11.44027 | 1.271141 |  |  |
| Exch. S | no^a^ | species | 1 | 0.569 | 0.569 | 0.332 | 0.581 |
|  |  | residuals | 8 | 13.72 | 1.715 |  |  |
| Exch. Zn | no | species | 1 | 4169.4 | 4169.4 | 1.321 | 0.280 |
|  |  | residuals | 9 | 28413 | 3157 |  |  |
| Extr. Al | no | species | 1 | 1415 | 1415 | 0.200 | 0.665 |
|  |  | residuals | 10 | 70928 | 7093 |  |  |
| Extr. B | rank^b^ | species | 1 | 0.0857 | 0.0857 | 0.006 | 0.940 |
|  |  | residuals | 10 | 143 | 14.3 |  |  |
| Extr. Ca | log | species | 1 | 0.0159 | 0.0159 | 0.042 | 0.842 |
|  |  | residuals | 10 | 3.8180 | 0.3818 |  |  |
| Extr. Cd | no | species | 1 | 2.37 | 2.37 | 0.209 | 0.657 |
|  |  | residuals | 10 | 113.41 | 11.34 |  |  |
| Extr. Cr | no | species | 1 | 0.0000957 | 0.0000957 | 0.104 | 0.754 |
|  |  | residuals | 10 | 0.0091923 | 0.0009192 |  |  |
| Extr. Cu | no | species | 1 | 0.1728 | 0.1728 | 0.023 | 0.882 |
|  |  | residuals | 10 | 74.1 | 7.41 |  |  |
| Extr. Fe | no | species | 1 | 2723 | 2723 | 2.307 | 0.160 |
|  |  | residuals | 10 | 11804 | 1180 |  |  |
| Extr. K | no | species | 1 | 0.489 | 0.489 | 0.001 | 0.980 |
|  |  | residuals | 10 | 7092 | 709 |  |  |
| Extr. Mg | log | species | 1 | 0.0558 | 0.0558 | 0.144 | 0.713 |
|  |  | residuals | 10 | 3.8869 | 0.3887 |  |  |
| Extr. Mn | no | species | 1 | 23.6 | 23.6 | 0.195 | 0.668 |
|  |  | residuals | 10 | 1211 | 121 |  |  |
| Extr. Ni | no | species | 1 | 0.000884 | 0.000884 | 0.113 | 0.744 |
|  |  | residuals | 10 | 0.078512 | 0.007851 |  |  |
| Extr. P | log | species | 1 | 0.185 | 0.185 | 1.106 | 0.318 |
|  |  | residuals | 10 | 1.675 | 0.167 |  |  |
| Extr. Pb | no | species | 1 | 4717 | 4717 | 0.047 | 0.832 |
|  |  | residuals | 10 | 997994 | 99799 |  |  |
| Extr. S | log | species | 1 | 0.0197 | 0.0197 | 0.082 | 0.780 |
|  |  | residuals | 10 | 2.4036 | 0.2404 |  |  |
| Extr. Zn | no | species | 1 | 6171 | 6171 | 0.187 | 0.675 |
|  |  | residuals | 10 | 330039 | 33004 |  |  |
| H | log | species | 1 | 0.986 | 0.986 | 0.458 | 0.514 |
|  |  | residuals | 10 | 21.5 | 2.15 |  |  |

^a^outlier removed (> mean + 2 x SD); ^b^rank transformed with rank() function in R setting ties.method to random

| (*b*) ANOVA of the linear model Response variable ~ species for the Klet site | | | | | | | |
| --- | --- | --- | --- | --- | --- | --- | --- |
| **Response variable** | **transformation of  response variable** |  | ***df*** | **SS** | **MS** | ***F*** | ***P*** |
| Exch. Al | no | species | 1 | 1.57 | 1.57 | 1.057 | 0.319 |
|  |  | residuals | 16 | 23.8 | 1.5 |  |  |
| Exch. B | no | species | 1 | 0.000068 | 0.000068 | 0.127 | 0.726 |
|  |  | residuals | 16 | 0.00862 | 0.00054 |  |  |
| Exch. Ca | no | species | 1 | 622535 | 622535 | 4.176 | 0.058 |
|  |  | residuals | 16 | 2385406 | 149087.9 |  |  |
| Exch. Cd | log | species | 1 | 1.13 | 1.13 | 1.713 | 0.209 |
|  |  | residuals | 16 | 10.57 | 0.66 |  |  |
| Exch. Cr | no | species | 1 | 0.00000014 | 0.00000014 | 0.027 | 0.870 |
|  |  | residuals | 16 | 0.0000798 | 0.0000050 |  |  |
| Exch. Cu | log | species | 1 | 0.777 | 0.777 | 1.454 | 0.245 |
|  |  | residuals | 16 | 8.55 | 0.53 |  |  |
| Exch. Fe | no | species | 1 | 5.00 | 5.00 | 0.730 | 0.405 |
|  |  | residuals | 16 | 109.49 | 6.84 |  |  |
| Exch. K | log | species | 1 | 0.236 | 0.236 | 1.475 | 0.242 |
|  |  | residuals | 16 | 2.556 | 0.160 |  |  |
| Exch. Mg | no | species | 1 | 726 | 726 | 1.275 | 0.275 |
|  |  | residuals | 16 | 9111 | 569 |  |  |
| Exch. Mn | no | species | 1 | 10.5 | 10.5 | 0.181 | 0.676 |
|  |  | residuals | 16 | 928.7 | 58.0 |  |  |
| Exch. Ni | no | species | 1 | 0.000115 | 0.000115 | 1.017 | 0.328 |
|  |  | residuals | 16 | 0.001810 | 0.000113 |  |  |
| Exch. P | log | species | 1 | 0.289 | 0.289 | 1.671 | 0.215 |
|  |  | residuals | 16 | 2.765 | 0.173 |  |  |
| Exch. Pb | log | species | 1 | 0.213 | 0.213 | 0.376 | 0.548 |
|  |  | residuals | 16 | 9.058 | 0.566 |  |  |
| Exch. S | log | species | 1 | 0.0324 | 0.0324 | 0.173 | 0.683 |
|  |  | residuals | 16 | 2.9979 | 0.1874 |  |  |
| Exch. Zn | log | species | 1 | 2.36 | 2.36 | 1.659 | 0.216 |
|  |  | residuals | 16 | 22.80 | 1.42 |  |  |
| Extr. Al | no | species | 1 | 74702 | 74702 | 4.930 | ***0.041*** |
|  |  | residuals | 16 | 242452 | 15153 |  |  |
| Extr. B | rank^b^ | species | 1 | 0.0857 | 0.0857 | 0.006 | 0.940 |
|  |  | residuals | 10 | 142.9 | 14.3 |  |  |
| Extr. Ca | log | species | 1 | 0.493 | 0.493 | 3.155 | 0.095 |
|  |  | residuals | 16 | 2.501 | 0.156 |  |  |
| Extr. Cd | log | species | 1 | 0.0205 | 0.0205 | 0.058 | 0.813 |
|  |  | residuals | 16 | 5.677 | 0.355 |  |  |
| Extr. Cr | log | species | 1 | 0.105 | 0.105 | 1.881 | 0.189 |
|  |  | residuals | 16 | 0.8919 | 0.0557 |  |  |
| Extr. Cu | log | species | 1 | 1.838 | 1.838 | 5.514 | ***0.032*** |
|  |  | residuals | 16 | 5.333 | 0.333 |  |  |
| Extr. Fe | log | species | 1 | 1.876 | 1.876 | 4.315 | 0.054 |
|  |  | residuals | 16 | 6.957 | 0.435 |  |  |
| Extr. K | log | species | 1 | 0.149 | 0.149 | 0.699 | 0.415 |
|  |  | residuals | 16 | 3.399 | 0.212 |  |  |
| Extr. Mg | no^a^ | species | 1 | 320 | 320 | 0.212 | 0.652 |
|  |  | residuals | 15 | 22670 | 1511 |  |  |
| Extr. Mn | no | species | 1 | 156 | 156 | 0.012 | 0.913 |
|  |  | residuals | 16 | 201703 | 12606 |  |  |
| Extr. Ni | rank^b^ | species | 1 | 34.7 | 34.7 | 1.235 | 0.283 |
|  |  | residuals | 16 | 449.8 | 28.1 |  |  |
| Extr. P | log | species | 1 | 1.273 | 1.273 | 3.146 | 0.095 |
|  |  | residuals | 16 | 6.473 | 0.405 |  |  |
| Extr. Pb | log | species | 1 | 3.62 | 3.62 | 2.465 | 0.136 |
|  |  | residuals | 16 | 23.49 | 1.47 |  |  |
| Extr. S | log | species | 1 | 0.0301 | 0.0301 | 0.164 | 0.691 |
|  |  | residuals | 16 | 2.938 | 0.184 |  |  |
| Extr. Zn | log | species | 1 | 0.151 | 0.151 | 0.459 | 0.508 |
|  |  | residuals | 15 | 4.944 | 0.330 |  |  |
| H | log | species | 1 | 0.892 | 0.892 | 1.436 | 0.248 |
|  |  | residuals | 16 | 9.934 | 0.621 |  |  |

^a^outlier removed (> mean + 2 x SD); ^b^rank transformed with rank() function in R setting ties.method to random

| **Table S3.** Statistical analysis of soil data for EAA  (*a*) ANOVA of the linear model Response variable ~ site for *Arabidopsis halleri* | | | | | | | |
| --- | --- | --- | --- | --- | --- | --- | --- |
| **Response variable** | **transformation of  responsive value** |  | ***df*** | **SS** | **MS** | ***F*** | ***P*** |
| Exch. Cd | Box-Cox | site | 3 | 1.50015 | 0.500005 | 46.748 | 1.03*10^-9^ |
|  |  | residuals | 22 | 0.23533 | 0.0107 |  |  |
| Exch. Zn | Box-Cox | site | 3 | 1.43166 | 0.47722 | 13.447 | 3.36*10^-5^ |
|  |  | residuals | 22 | 0.78074 | 0.03549 |  |  |

| (*b*) ANOVA of the linear model Response variable ~ site for *Arabidopsis arenosa* | | | | | | | | | | | | | |  |
| --- | --- | --- | --- | --- | --- | --- | --- | --- | --- | --- | --- | --- | --- | --- |
| **Response variable** | | **transformation of  response variable** |  | | ***df*** | | **SS** | | **MS** | | ***F*** | | ***P*** |  |
| Exch. Cd | Box-Cox | | | site | | 3 | | 2.69805 | | 089935 | | 149.73 | <2.2*10^-16^ | |
|  |  | | | residuals | | 26 | | 0.15616 | | 0.00601 | |  |  | |
| Exch. Zn | Box-Cox | | | site | | 3 | | 0.090801 | | 0.030267 | | 68.63 | 1.75*10^-12^ | |
|  |  | | | residuals | | 26 | | 0.01146 | | 0.000441 | |  |  | |

| (c) Summary of the linear model Response variable ~ site for *Arabidopsis halleri* | | | | | |
| --- | --- | --- | --- | --- | --- |
| **Response variable** | **Factor level** | **Estimate** | **Std. Error** | ***t value*** | ***P*** |
| Exch. Cd | (Intercept) | 0.5518 | 0.04625 | 11.93 | 4.45*10^-11^ |
|  | Site Zako | 0.06206 | 0.06056 | 1.025 | 0.317 |
|  | Site Klet | 0.36527 | 0.05769 | 6.332 | 2.26*10^-6^ |
|  | Site Mias | 0.65763 | 0.06541 | 10.054 | 1.09*10^-9^ |
| Exch. Zn | (Intercept) | 1.08788 | 0.08425 | 12.913 | 9.6*10^-12^ |
|  | Site Zako | -0.12115 | 0.11031 | -1.098 | 0.283958 |
|  | Site Klet | 0.2397 | 0.10508 | 2.281 | 0.032572 |
|  | Site Mias | 0.5327 | 0.11914 | 4.471 | 0.000191 |

Factor levels refer to ‘site’. The non-metalliferous site Kowa was set as the base factor level (intercept).

| (d) Summary of the linear model Response variable ~ site for *Arabidopsis arenosa* | | | | | |
| --- | --- | --- | --- | --- | --- |
| **Response variable** | **Factor level** | **Estimate** | **Std. Error** | ***t value*** | ***P*** |
| Exch. Cd | (Intercept) | 1.66543 | 0.0274 | 60.78 | < 2*10^-16^ |
|  | Site Zapa | -0.05774 | 0.03875 | -1.49 | 0.148 |
|  | Site Klet | -0.5388 | 0.03766 | -14.31 | 7.75*10^-14^ |
|  | Site Mias | -0.74618 | 0.04418 | -16.89 | 1.56*10^-15^ |
| Exch. Zn | (Intercept) | 0.954992 | 0.007425 | 128.623 | < 2*10^-16^ |
|  | Site Zapa | 0.003181 | 0.0105 | 0.303 | 0.764 |
|  | Site Klet | 0.086432 | 0.010204 | 8.47 | 5.96*10^-9^ |
|  | Site Mias | 0.140908 | 0.011972 | 11.77 | 6.43*10^-12^ |

Factor levels refer to ‘site’. The non-metalliferous site Kowa was set as the base factor level (intercept).

| (e) Descriptive statistics for response variables per site for *Arabidopsis halleri* | | | | | | | |
| --- | --- | --- | --- | --- | --- | --- | --- |
| **Response variable** | **Site** | **N** | **Mean** | **Median** | **Std. deviation** | **IQR** | **CV** |
| Exch. Cd | Kowa | 5 | 0.027 | 0.010 | 0.0409 | 0.0000 | 1.5135 |
|  | Zako | 7 | 0.045 | 0.040 | 0.0364 | 0.0550 | 0.8089 |
|  | Klet | 9 | 0.742 | 0.690 | 0.6625 | 0.4800 | 0.8926 |
|  | Mias | 5 | 4.046 | 3.800 | 1.5223 | 0.5600 | 0.3762 |
| Exch. Zn | Kowa | 5 | 29.1680 | 0.8500 | 63.5739 | 0.6600 | 2.1796 |
|  | Zako | 7 | 1.1057 | 1.4100 | 0.7672 | 1.1500 | 0.6938 |
|  | Klet | 9 | 27.0989 | 17.3000 | 24.1239 | 41.4800 | 0.8902 |
|  | Mias | 5 | 127.1220 | 129.3600 | 49.9702 | 64.8500 | 0.3931 |

IQR – interquartile range, CV – coefficient of variance

| (f) Descriptive statistics for response variables per site for *Arabidopsis arenosa* | | | | | | | |
| --- | --- | --- | --- | --- | --- | --- | --- |
| **Response variable** | **Site** | **N** | **Mean** | **Median** | **Std. deviation** | **IQR** | **CV** |
| Exch. Cd | Kowa | 8 | 0.0075 | 0.0050 | 0.0053 | 0.0013 | 0.7127 |
|  | Zapa | 8 | 0.0100 | 0.0100 | 0.0046 | 0.0013 | 0.4629 |
|  | Klet | 9 | 0.3622 | 0.3300 | 0.2046 | 0.3600 | 0.5649 |
|  | Mias | 5 | 3.6500 | 2.3000 | 3.7954 | 3.1200 | 1.0398 |
| Exch. Zn | Kowa | 8 | 0.2763 | 0.0500 | 0.5267 | 0.1125 | 1.9067 |
|  | Zapa | 8 | 0.1600 | 0.1200 | 0.1352 | 0.1525 | 0.8452 |
|  | Klet | 9 | 11.5022 | 5.9500 | 11.2786 | 13.9400 | 0.9806 |
|  | Mias | 5 | 117.8400 | 131.9800 | 71.4932 | 64.3100 | 0.6067 |

IQR – interquartile range, CV – coefficient of variance

| (*a*) Results (see figure 2) | | | | | | | | | |
| --- | --- | --- | --- | --- | --- | --- | --- | --- | --- |
|  |  |  | Control-soil | | |  | M-soil | |  |
| Species | Population | Genotype | Number of plants | Biomass^*^ [g] Mean ± SD | Survival rate |  | Number of plants | Biomass^*^ [g] Mean ± SD | Survival rate |
| *A. halleri* | Miasteczko | Mias_01 | 3 | 0.70 ± 0.43 | 1 |  | 3 | 0.56 ±0.15 | 1 |
|  | Śląskie | Mias_03 | 3 | 1.85 ± 0.05 | 1 |  | 3 | 0.83 ±0.21 | 1 |
|  |  | Mias_05 | 3 | 1.28 ± 0.87 | 1 |  | 3 | 1.41 ±0.06 | 1 |
|  |  | Mias_09 | 2 (1) | 0.89 | 1 |  | 2 | 0.82 ±0.61 | 1 |
|  | Zakopane | Zako_03 | 3 (1) | 1.72 | 1 |  | 3 (2) | 0.60 ±0.22 | 1 |
|  |  | Zako_09 | 3 (1) | 0.92 | 1 |  | 3 | 0.32 ±0.10 | 1 |
|  |  | Zapa_03 | 3 (2) | 1.95 ± 0.47 | 1 |  | 3 | 0.52 ±0.06 | 1 |
|  |  | Zapa_11 | 3 | 1.51 ± 0.54 | 1 |  | 3 | 0.72 ±0.14 | 1 |
| *A. arenosa* | Miasteczko | Mias_001_a01 | 3 | 2.59 ± 0.22 | 1 |  | 3 | 0.76 ± 0.58 | 1 |
|  | Śląskie | Mias_001_a04 | 3 | 2.59 ± 0.35 | 1 |  | 3 (2) | 1.11 ± 0.14 | 1 |
|  | Zakopane | Zapa_001_a01 | 3 (2) | 2.04 ± 0.06 | 1 |  | 3 | 0.1 ± 0 | 0 |
|  |  | Zapa_001_a02 | 3 (2) | 1.23 ± 0.16 | 1 |  | 3 | 0.1 ± 0 | 0 |
|  |  | Zapa_001_a03 | 1 | 1.96 | 1 |  | 1 | 0.1 | 0 |
|  |  | Zapa_001_a04 | 3 | 1.48 ± 0.10 | 1 |  | 3 | 0.1 ± 0 | 0 |
| ^*^ Fresh biomass  Numbers in brackets are numbers used for the analysis: plants that looked sick directly after transferring to treatment soil or showed reduction in biomass on control soil were removed. Dead plants biomass were set to 0.1 g. | | | | | | | | | |

**Table S4**. Results of the local adaptation experiments including statistical analysis

| (*b*) Generalized linear mixed effect model (see figure 2) | | | | | | |
| --- | --- | --- | --- | --- | --- | --- |
|  | ***A. arenosa*** | | | ***A. halleri*** | | |
| **Response variable: Fresh biomass** | *df* | χ ^2^ | *P* | *df* | χ ^2^ | *P* |
| Genotype | 1 | 4.0196 | **0.045** | 1 | 0.2042 | 0.65 |
| Treatment | 1 | 13.9132 | **<0.001** | 1 | 5.8631 | **0.016** |
| Genotype:Treatment | 1 | 205.6942 | **<0.001** | 1 | 6.1597 | **0.013** |
| Significant p-values are in bold (*P* < 0.05).  Genotype = site of origin; Mias metalliferous site vs. Zapa non-metalliferous site Treatment = experimental soil type of exposure; control-soil vs. metalliferous soil | | | | | | |

| (*c*) Results (see figure S3) | | | | | | | | | |
| --- | --- | --- | --- | --- | --- | --- | --- | --- | --- |
|  |  |  | Control-soil | | |  | M-soil | |  |
| Species | Population | Genotype | Number of plants | Biomass^*^ [g] Mean ± SD | Survival rate |  | Number of plants | Biomass^*^ [g] Mean ± SD | Survival rate |
| *A. halleri* | Miasteczko | Mias_01 | 4 | 0.95 ± 0.64 | 1 |  | 4 | 0.93 ± 0.17 | 1 |
|  | Śląskie | Mias_03 | 4 | 1.98 ± 0.61 | 1 |  | 3 | 0.90 ± 0.3 | 1 |
|  |  | Mias_05 | 1(0) | NA | NA |  | 1 (0) | NA | NA |
|  |  | Mias_09 | 1 | 2.40 | 1 |  | 2 (1) | 1.40 | 1 |
|  | Zakopane | Zako_02 | 2 (1) | 0.40 | 1 |  | 2 | 0.95 ± 1.20 | 0.5 |
|  |  | Zako_03 | 2 (1) | 2.10 | 1 |  | 2 | 0.55 ± 0.35 | 1 |
|  |  | Zako_05 | 1 (0) | NA | NA |  | 1 | 0.60 | 1 |
|  |  | Zako_06 | 1 (0) | NA | NA |  | 1 | 0.40 | 1 |
|  |  | Zako_09 | 2 (1) | 1.80 | 1 |  | 2 | 0.90 ± 0.42 | 1 |
|  |  | Zako_10 | 2 (1) | 3.50 | 1 |  | 2 | 1.80 ± 0 | 1 |
|  |  | Zapa_01 | 4 (3) | 1.27 ± 0.21 | 1 |  | 4 | 0.48 ± 0.10 | 1 |
|  |  | Zapa_07 | 4 | 1.38 ± 0.73 | 1 |  | 4 | 0.70 ± 0.18 | 1 |
|  |  | Zapa_11 | 4 | 2.58 ± 1.20 | 1 |  | 4 | 0.88 ± 0.40 | 1 |
| *A. arenosa* | Miasteczko | Mias_001_a10 | 3 | 3.63 ± 0.95 | 1 |  | 3 | 0.87 ± 0.75 | 0.67 |
|  | Śląskie | Mias_003_a11 | 3 (2) | 2.85 ± 0.92 | 1 |  | 2 | 0.10 ± 0 | 0 |
|  |  | Mias_001_a16 | 3 | 2.67 ± 1.79 | 1 |  | 3 | 1.47 ± 2.25 | 1 |
|  |  | Mias_001_a17 | 2 (1) | 2.60 | 1 |  | 2 (1) | 1.60 | 1 |
|  |  | Mias_001_a18 | 1 | 2.80 | 1 |  | 1 | 0.10 | 0 |
|  | Zakopane | Zapa_001_a08 | 1 | 2.00 | 1 |  | 2 | 0.10 ± 0 | 0 |
|  |  | Zapa_001_a11 | 3 | 1.47 ± 0.46 | 1 |  | 3 | 0.10 ± 0 | 0 |
| ^*^ Fresh biomass  Numbers in brackets are numbers used for the analysis: plants that looked sick directly after transferring to treatment soil or showed reduction in biomass on control soil were removed. Dead plants biomass were set to 0.1 g. | | | | | | | | | |

| (*d*) Generalized linear mixed effect model (see figure S3) | | | | | | |
| --- | --- | --- | --- | --- | --- | --- |
|  | ***A. arenosa*** | | | ***A. halleri*** | | |
| **Response variable: Fresh biomass** | *df* | χ ^2^ | *P* | *df* | χ ^2^ | *P* |
| Genotype | 1 | 0.3139 | 0.58 | 1 | 0.0033 | 0.95 |
| Treatment | 1 | 2.1548 | 0.14 | 1 | 9.3996 | **0.002** |
| Genotype:Treatment | 1 | 10.1404 | **0.0015** | 1 | 0.7986 | 0.37 |
| Significant p-values are in bold (*P* < 0.05).  Genotype = site of origin; Mias metalliferous site vs. Zapa non-metalliferous site Treatment = experimental soil type of exposure; control-soil vs. metalliferous soil | | | | | | |

**Table S5.** Hypergeometric tests for the number of overlapping genes expected by chance.

(*a*) Based on all genes of the *A. lyrata* reference genome*

| *p*-values | *arenosa*  Klet-Kowa | *halleri*  Mias-Zapa | *halleri*  Klet-Kowa |
| --- | --- | --- | --- |
| *arenosa* Mias-Zapa | **0.0003** | 0.056 | 0.26 |
| *arenosa* Klet-Kowa |  | **0.009** | 0.28 |
| *halleri* Mias-Zapa |  |  | **2 x 10^-6^** |

* 33,221 genes based on *A. lyrata* annotation version 2

(*b*) Based on an approximated number of 25,000 genes shared between species

| *p*-values | *arenosa*  Klet-Kowa | *halleri*  Mias-Zapa | *halleri*  Klet-Kowa |
| --- | --- | --- | --- |
| *arenosa* Mias-Zapa | **0.001** | 0.092 | 0.32 |
| *arenosa* Klet-Kowa |  | **0.02** | 0.35 |
| *halleri* Mias-Zapa |  |  | **8 x 10^-6^** |

We tested the likelihood of obtaining the observed or a larger number of genes by chance for each intersecting gene set (*a*, *b*; see figure 3).
