# Supplementary material for: Convergent evolution in Arabidopsis halleri and Arabidopsis arenosa on calamine metalliferous soils
Source: Philos Trans R Soc Lond B Biol Sci. 2019 Jun 3;374(1777):20180243. doi: 10.1098/rstb.2018.0243 (PMC6560266; doi:10.1098/rstb.2018.0243)
Supplement: Figure S7 [file rstb20180243supp7.pdf]

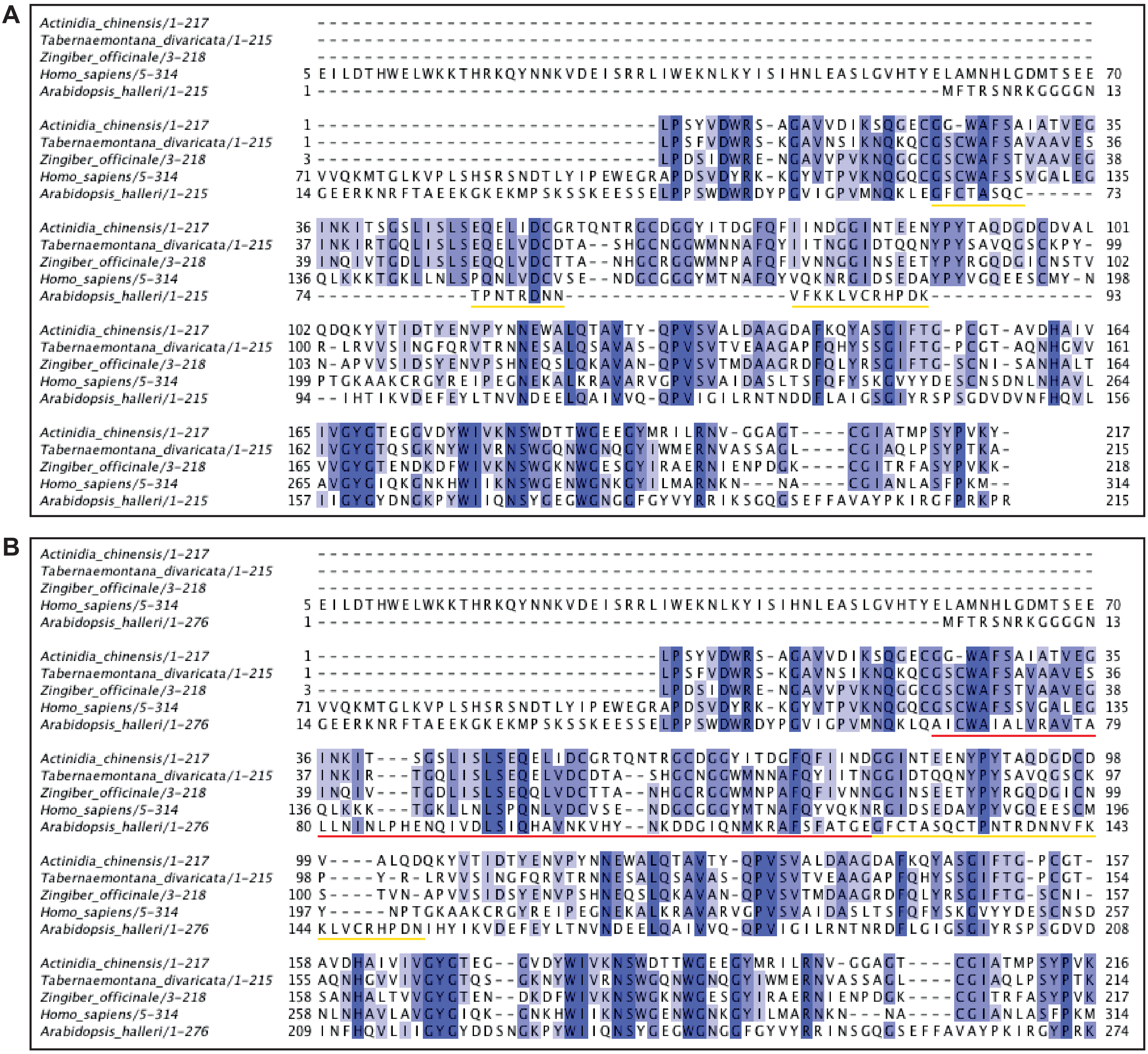

Figure S7. Multiple sequence alignment (MSA) of Papain-like cysteine proteases. Shown are *Arabidopsis halleri* (*Ah*CPL1), *Actinidia chinensis* (PDB: 2ACT), *Tabernaemontana divaricata* (PDB: 1IWD), *Zingiber officinale* (PDB: 1CQD) and *Homo sapiens* (PDB: 1BY8). **A**, MSA showing the *Ah*CPL1 protein generated by mapping to *A. lyrata* exons. **B**, MSA showing the *Ah*CPL1 protein generated based on *A. halleri* exons. Sequence incorperated based on the genome of *A. halleri* instead of *A. lyrata* is underlined in red. Sequence repositioned based on *A. halleri* sequence data is underlined in yellow.
