# Supplementary material for: Convergent evolution in Arabidopsis halleri and Arabidopsis arenosa on calamine metalliferous soils
Source: Philos Trans R Soc Lond B Biol Sci. 2019 Jun 3;374(1777):20180243. doi: 10.1098/rstb.2018.0243 (PMC6560266; doi:10.1098/rstb.2018.0243)
Supplement: Figure S8 [file rstb20180243supp8.pdf]

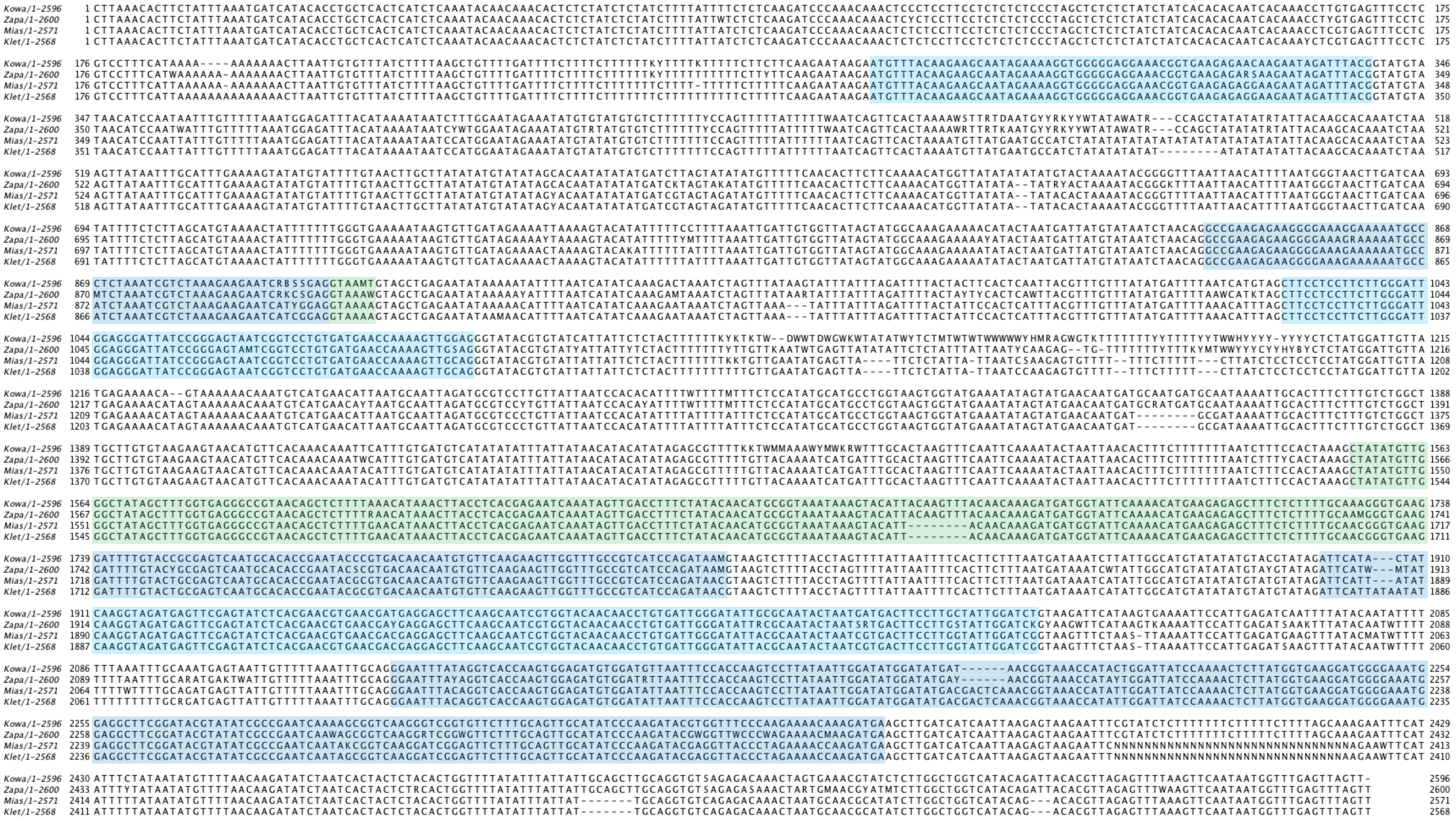

Figure S8. Multiple sequence alignment of consensus genomic DNA sequence at the AhCPL1 locus. Probable coding exons are highlighted. Sequence incorperated based on the *A. lyrata* genomic assembly are highlighted in in alternating shades of blue. Additional exonic sequence incorporated from the *A.halleri* genome is highlighted in greeen
